# Human glutaredoxin-1 can transfer copper to isolated metal binding domains of the P_1B_-type ATPase, ATP7B

**DOI:** 10.1038/s41598-020-60953-z

**Published:** 2020-03-05

**Authors:** Shadi Maghool, Sharon La Fontaine, Blaine R. Roberts, Ann H. Kwan, Megan J. Maher

**Affiliations:** 1 Department of Biochemistry and Genetics, La Trobe Institute for Molecular Science, La Trobe University, Melbourne, VIC Australia; 2 School of Life and Environmental Sciences, Deakin University, Geelong, VIC Australia; 3 The Florey Institute of Neuroscience, The University of Melbourne, Parkville, VIC Australia; 4 Department of Biochemistry, Emory University School of Medicine, Atlanta, GA 30322 USA; 5 School of Life and Environmental Sciences and University of Sydney Nano Institute, Sydney, NSW Australia; 6 School of Chemistry and The Bio21 Molecular Science and Biotechnology Institute, The University of Melbourne, Parkville, Australia

**Keywords:** Ion transport, Biophysical chemistry

## Abstract

Intracellular copper (Cu) in eukaryotic organisms is regulated by homeostatic systems, which rely on the activities of soluble metallochaperones that participate in Cu exchange through highly tuned protein-protein interactions. Recently, the human enzyme glutaredoxin-1 (hGrx1) has been shown to possess Cu metallochaperone activity. The aim of this study was to ascertain whether hGrx1 can act in Cu delivery to the metal binding domains (MBDs) of the P_1B_-type ATPase ATP7B and to determine the thermodynamic factors that underpin this activity. hGrx1 can transfer Cu to the metallochaperone Atox1 and to the MBDs 5-6 of ATP7B (WLN5-6). This exchange is irreversible. In a mixture of the three proteins, Cu is delivered to the WLN5-6 preferentially, despite the presence of Atox1. This preferential Cu exchange appears to be driven by both the thermodynamics of the interactions between the proteins pairs and of the proteins with Cu(I). Crucially, protein-protein interactions between hGrx1, Atox1 and WLN5-6 were detected by NMR spectroscopy both in the presence and absence of Cu at a common interface. This study augments the possible activities of hGrx1 in intracellular Cu homeostasis and suggests a potential redundancy in this system, where hGrx1 has the potential to act under cellular conditions where the activity of Atox1 in Cu regulation is attenuated.

## Introduction

Copper (Cu) is a redox active metal and an essential trace element for human health^1,2^. In biological systems, Cu is found in two oxidation states, reduced Cu(I) (cuprous) and oxidized Cu(II) (cupric). These reversible states, which allow Cu to serve as both an oxidant and reductant, render it an exceptional cofactor for redox enzymes known as oxidoreductases^1,2^, however excess intracellular concentrations of Cu are toxic and lead to cellular oxidative damage^3,4^. In particular, the reaction of hydrogen peroxide with Cu(I) and the reduction of Cu(II) by superoxide, produce hydroxyl radicals that damage proteins, nucleic acids and lipids^5,6^.

In humans, the dysregulation of Cu metabolism is associated with diseases such as Menkes syndrome, Wilson’s disease, prion diseases, Alzheimer’s disease and fatal motor neuron diseases such as familial amyotrophic lateral sclerosis^7–12^. To maintain the fine balance between cellular Cu requirements and toxicity, organisms have evolved sophisticated metalloregulatory systems for the coordination of metals such as Cu to proteins within the cell, and the controlled transfer of these metals between protein partners. Soluble proteins that are responsible for Cu binding and transfer are termed ‘metallochaperones’^3^, which transport Cu in its reduced state (Cu(I)), often *via* coordination with cysteine residues^13,14^. In eukaryotes, including humans, Cu concentrations are regulated through control of the levels of the Cu import protein, copper transporter 1 (Ctr1)^15,16^ at the plasma membrane and the trafficking and/or activity of the Cu export proteins, ATP7A or ATP7B^17–19^.

In humans, ATP7A (Menkes disease protein, MNK) and ATP7B (Wilson disease protein, WLN) are two Cu(I)-specific P_1B_-type ATPases that are essential for Cu transport and homeostasis. These ion pumps, which are localized in the *trans*-Golgi network (TGN), utilize ATP hydrolysis to transport the metal across the TGN membrane to activate Cu-dependent enzymes within the secretory pathway (eg. ceruloplasmin)^17–19^. In addition, in response to elevated levels of intracellular Cu, these enzymes traffic from the TGN towards the cell periphery to export the excess Cu from the cell^19,20^. Crucially, only the metallochaperone Atox1 has been implicated in facilitating Cu delivery to the ATP7A and ATP7B proteins.

The ATP7A and ATP7B proteins share high levels of sequence similarity with other P_1B_-type ATPases, possessing an A-domain and an ATP-binding domain, which mediate their catalytic activities. The latter comprises the P-domain that includes the site of catalytic phosphorylation and the signature motifs for the P-type ATPases (DKTG, TGDN, GDGXND, where X is any amino acid), and the N (nucleotide binding)-domain^17,21–23^. However, a distinguishing feature of the ATP7A and ATP7B proteins is the presence of a large amino-terminal extension that is composed of six Cu-binding domains (~600 residues in total). These metal binding domains (MBDs) have ferredoxin-like βαββαβ folds and bind Cu(I) at C-XX-C motifs, which redox cycle between oxidized (disulfide) and reduced (thiol) states^7,24–33^. The MBDs participate in Cu exchange *via* protein-protein interactions, which are facilitated by flexible polypeptide linkers that bridge the domains^34,35^.

The mechanism by which the MBDs act to facilitate Cu delivery to the transmembrane domain (TMD) remains under investigation, with consensus within the literature still to be achieved. The ATP7B MBDs 1-4 were shown to be actively involved in receiving Cu from Atox1^27,36^ and an NMR titration experiment comparing Cu transfer from Atox1 to the protein constructs ATP7B MBD 4 and MBDs 5-6 indicated that Atox1 cannot transfer Cu to the MBD 5-6 protein, whereas Atox1 can participate in Cu exchange with the MBD 4 protein^21^. This study proposed a model for Cu binding and transport by ATP7B, where the MBD 4 receives Cu from Atox1 and passes the metal to MBD 6, which transfers Cu to MBD 5 for delivery to the TMD for translocation across the membrane^21^. In this way, the ATP7B MBDs 1-4 were suggested to play crucial roles in receipt of Cu by the transporter and in regulation of Cu transport, whereas the MBDs 5-6 function in passing Cu to the TMD of the transporter for efflux. However, more recent studies have established that the ATP7B MBD 4 does not interact with the other ATP7B MBDs, which has led to the proposal that this domain serves only as a structural link between the MBDs 1–3 (regulatory) and MBDs 5-6 (Cu-delivery)^21,35,37,38^.

Glutaredoxin-1 (Grx1) is a cytosolic member of a class of glutaredoxin enzymes, which are GSH-dependent thiol-disulfide oxidoreductases. Grx1 plays a significant role in the maintenance of cellular redox homeostasis *via* the catalysis of reversible thiol-disulfide exchange reactions between protein thiols and the substrates, namely reduced and oxidized glutathione (GSH and GSSG, respectively)^36,39,40^. The crystal structure of human glutaredoxin-1 (hGrx1) has been determined (PDB 4RQR)^41^ and was shown to adopt a thioredoxin fold^42^, with an exposed C-XX-C motif (C23-XX-C26; residues numbered according to UniProtKB P35754)^43^. This motif was shown by site-directed mutagenesis and related analyses to be the active site of the enzyme and also the high affinity Cu(I) binding site^10,44,45^. Studies using yeast two-hybrid and mammalian co-immunoprecipitation experiments demonstrated that hGrx1 interacts with the MBDs of the ATP7A and ATP7B proteins in a Cu-dependent manner^46,47^. Furthermore, additional reports that described the overexpression and knocking down of hGrx1 in neuronal, non-neuronal cells and the generation and analysis of Grx1^KO^ mouse embryonic fibroblast (MEF) cells, showed impact specifically on Cu homeostasis^36,47,48^, which may have resulted from the redox or Cu-binding activities (or both) of Grx1^49,50^.

hGrx1 has been shown to catalyze the reduction of a protein disulfide bond in the human Cu,Zn superoxide dismutase at the expense of GSH^51^ and mediate the oxidation and reduction of Atox1 with the direction of catalysis dependent on the cytoplasmic GSSG/GSH ratio and Cu availability^10^. Specifically, the latter study showed that in the presence of Cu, hGrx1 could facilitate the reduction of oxidized Atox1 using GSH as a substrate, while the reverse reaction (oxidation of Atox1), catalyzed by hGrx1 (with GSSG as a substrate) was Cu independent^10^. Analyses of the recombinant hGrx1 protein also demonstrated that hGrx1 binds Cu(I) with femtomolar affinity (*K*_DCu(I)_ ~ 10^–15^ M) *via* its active site Cys residues, but that Cu(I) binding inhibits hGrx1 enzyme activity^10^, potentially alluding to a mechanism for the regulation of the enzyme activity that is dependent on the intracellular reduction potential and availability of Cu(I). These data are consistent with reports that demonstrate a role for hGrx1 in Cu metabolism and in protecting neuronal cells from Cu toxicity^36,48^.

Research seeking to establish the driving factors for Cu delivery to its cellular destinations has to date focused almost exclusively on the determination of the relative Cu-binding affinities of each individual protein partner^1,44^. Consistently, the binding affinities of these proteins for Cu(I) have been characterized as exceptionally tight with *K*_DCu(I)_ values in the range 10^−15^–10^−17^ M^10,44^. With such high affinity binding, the question of how Cu transfer from one protein to another is achieved has led to the hypothesis that affinity gradients between proteins determine the direction of Cu exchange^1^. Few structures of a protein complex in the act of Cu-transfer have so far been determined. The structures of a complex of the Atx1 and Ccc2a proteins from *Saccharomyces cerevisae*^52^ and that between Atox1 and the ATP7A MBD1 have been described by NMR^53^, both of which show a Cu(I)-bridged assembly, with the Cu(I) atom coordinated by a pair of Cys residues from each protein. Despite these structural characterizations, the thermodynamics of the interactions between the two protein partners were not reported. However, these interactions were described as being Cu-dependent^52^.

In the current study, we have investigated the protein-protein interactions between hGrx1, and the proteins Atox1 and ATP7B MBDs 5-6 (hereafter referred to as WLN5-6), both in Cu-bound and *apo* (Cu-free) states, in conjunction with their intermolecular Cu-transfer activities. These data show that the protein-protein interactions are not Cu-dependent and that the affinities of the protein-protein interactions differ between pairs of protein partners. We therefore propose that the affinities of protein-protein interactions in partnership with the Cu(I)-binding affinities of the individual proteins determine Cu trafficking within the cell. Critically, we establish a potential Atox1-independent mechanism for Cu delivery to ATP7B, through the action of hGrx1, and therefore an alternative pathway for the maintenance of intracellular Cu homeostasis.

## Results and Discussion

### Recombinant hGrx1, Atox1 and WLN5-6 proteins bind Cu(I) with sub-femtomolar affinities

Recombinant hGrx1, Atox1 and WLN5-6 proteins were overexpressed and purified (Fig. S1). All purified proteins, including Atox1 and WLN5-6, in addition to unlabeled and/or ^15^N- and ^13^C/^15^N-labelled hGrx1 were confirmed to be isolated as *apo*-proteins as determined by inductively coupled plasma mass spectrometry (ICP-MS) and a colorimetric assay with Bcs. Purified *apo*-proteins were loaded with Cu(I) as previously described^10^ and after size exclusion chromatography (SEC) to remove excess Cu(I), were analyzed for Cu(I) content. As expected, the Atox1 and hGrx1 proteins were found to bind Cu(I) with a Cu(I):protein stoichiometry of 1:1 while WLN5-6, which has two MBDs, bound Cu(I) with a stoichiometry of 2:1 (Table S1). The *apo*- and Cu(I)-bound proteins prepared in this way were used for both the copper-exchange and nuclear magnetic resonance (NMR) experiments.

To determine the binding affinities of the individual proteins for Cu(I), purified *apo*-proteins at various concentrations were added individually to a reaction mixture containing the probe complex [Cu^I^Bcs_2_]^3−^. These experiments yielded dissociation constants (*K*_DCu(I)_) for the hGrx1, Atox1, and WLN5-6 proteins of 10^−15.8^, 10^−17.5^ and 10^−17.8^ M at pH 7.0, respectively (Table S2). These determinations agree closely with those previously reported^10,44^ (Table S2).

### The hGrx1, Atox1 and WLN5-6 proteins participate in Cu(I)-transfer with *apo*-protein partners

**Figure 1:**
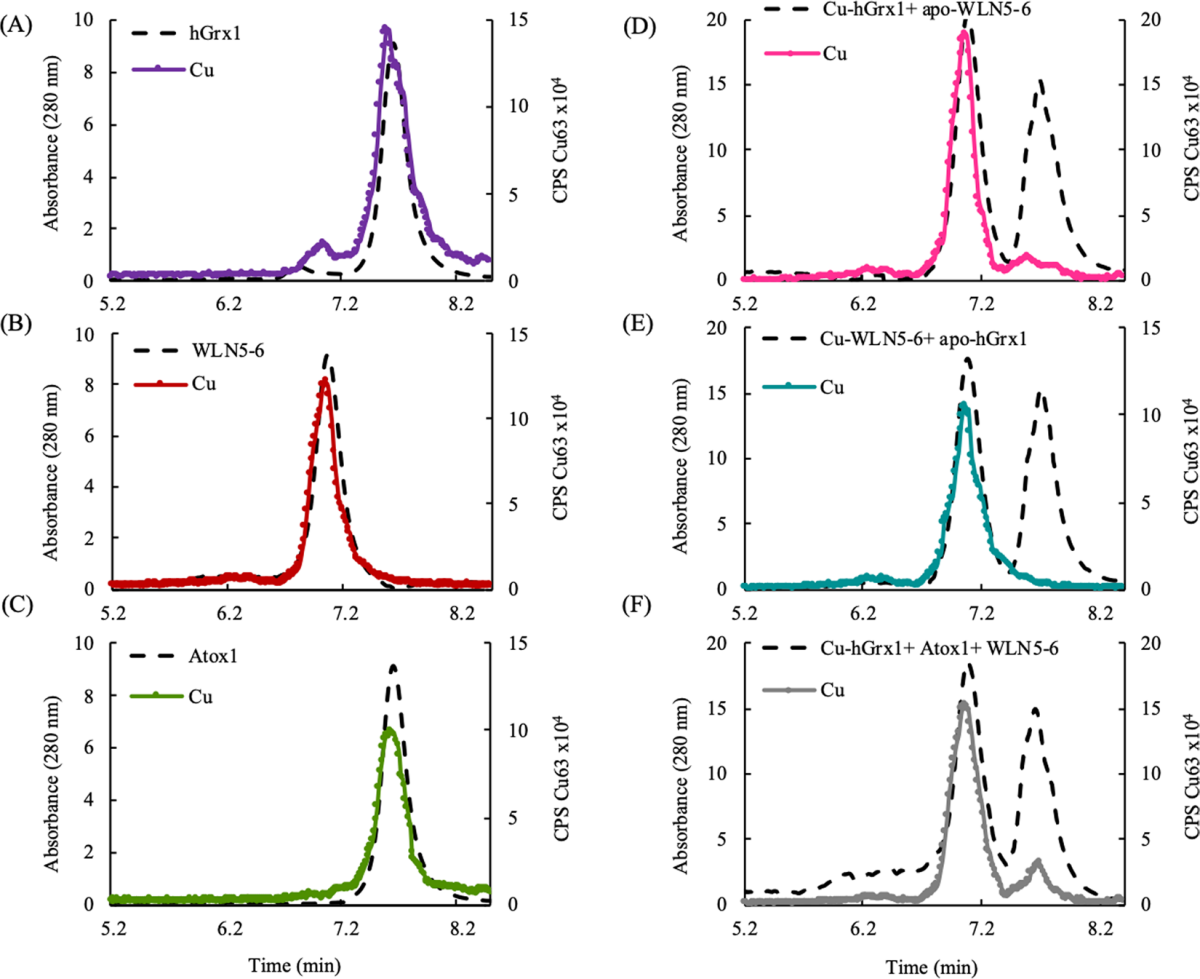
Result of Cu exchange reactions between Cu(I)-hGrx1 and partner proteins. (**A**) Cu(I)-hGrx1, (**B**) Cu(I)-WLN5-6 and (**C**) Cu(I)-Atox1 were applied to SEC column and fractions analyzed for the presence of protein (A_280_, dashed lines: black) and Cu (circles: hGrx1, purple; WLN5-6, red; Atox1, green) by ICP-MS. (**D**) Results of Cu exchange reaction between Cu(I)-hGrx1 and *apo*-WLN5-6. Cu(I)-hGrx1 and *apo*-WLN5-6 were incubated together at 1:1 molar and re-separated using SEC (A_280_, black dash lines). On separation, the Cu(I) elutes with the WLN5-6 protein, indicating Cu(I)-exchange (pink circles). (**E**) Results of Cu exchange reaction between Cu(I)-WLN5-6 and *apo*-hGrx1. The two proteins (*apo*-hGrx1 and Cu(I)-WLN5-6) were incubated together at 1:1 molar and re-separated the same technique (A_280_, black dash lines). On separation, the Cu(I) elutes with the WLN5-6 protein, indicating no Cu-exchange (teal circles). (**F**) Cu exchange between Cu(I)-hGrx1, and a mixture of the *apo*-Atox1 and *apo*-WLN5-6 proteins. Cu(I)-hGrx1, *apo*-Atox1 and *apo*-WLN5-6 were incubated together at 1:1:1 molar and separated using SEC (A_280_, black dash lines) and fractions analyzed for the presence of Cu by ICP-MS (gray circles).

To establish whether the hGrx1 and WLN5-6 proteins could interact to facilitate Cu exchange, we performed protein co-incubation assays followed by SEC-ICP-MS. The individual *apo*- and Cu(I)- proteins were incubated together before separation by SEC. The column eluent was simultaneously monitored for the presence of protein (A_280_) and for Cu (ICP-MS) (Fig. 1A–C). Co-incubation of the Cu(I)-hGrx1 and *apo*-WLN5-6 proteins yielded a SEC profile with two major protein peaks where the first peak (WLN5-6), co-eluted with Cu while the second peak (hGrx1) was essentially Cu-free (Fig. 1D), indicating Cu-transfer from Cu(I)-hGrx1 to *apo*-WLN5-6 to yield *apo*-hGrx1 and Cu(I)-WLN5-6. The reverse experiment (*apo*-hGrx1 with Cu(I)-WLN5-6) did not result in Cu exchange, indicating the former transfer was irreversible (Fig. 1E). In addition, through separation of the proteins by anion exchange and colorimetric analyses of the Cu(I) content of the eluents, we were able to reproduce the results of previous studies that showed irreversible Cu exchange between Cu(I)-hGrx1 and *apo*-Atox1 and no exchange between Cu(I)-Atox1 and *apo*-WLN5-6 or Cu(I)-WLN5-6 and *apo*-Atox1 (Figs. S2, S3)^10,21^.

This demonstrates that Cu(I)-hGrx1 is able to transfer Cu to both *apo*-protein partners Atox1 and WLN5-6. However, Cu was not observed to transfer from Cu(I)-Atox1 and Cu(I)-WLN5-6 to *apo*-hGrx1. This observation correlates with the significantly weaker Cu binding affinity of hGrx1 compared with the Atox1 and WLN5-6 proteins (Table S2)^1^. Crucially, the ability of Cu(I)-hGrx1 to transfer Cu to WLN5-6 has not been reported previously and augments the possible roles for hGrx1 in intracellular Cu homeostasis beyond Cu exchange with Atox1^10^.

The failure of the Atox1 and WLN5-6 proteins to participate in Cu-exchange in both directions is consistent with previously published studies and has been attributed to the inability of the proteins to interact productively for Cu transfer^21^. However, the observation of Cu transfer from Cu(I)-Atox1 to individual MDBs of ATP7B appears to vary depending on the experimental conditions^21,32,34,54^. Specifically, the WLN5 and WLN6 domains have been shown to receive Cu from Cu(I)-Atox1, both when present as individual domains in solution^54^ and as part of a multidomain (WLN1-6) protein fragment^34^.

### Transfer of Cu from Cu(I)-hGrx1 to WLN5-6 occurs despite the presence of Atox1

Our observations of pairwise Cu(I)-transfer between the hGrx1 and the *apo*-proteins Atox1 and WLN5-6 led us to extend the study to a mixture of all three of these proteins. Cu(I)-hGrx1 was incubated with a mixture of the *apo-*proteins Atox1 and WLN5-6 (at a molar ratio of 1:1:1), followed by re-separation by SEC-ICP-MS. Remarkably, we observed *via* SEC-ICP-MS that the majority of the Cu co-eluted with the WLN5-6 protein, rather than the hGrx1 and Atox1 proteins which, despite optimization of the conditions of the experiment, co-eluted as a single peak (Fig. 1F). The Cu(I) distribution was estimated (through the Cu(I) contents of the corresponding eluents, that is, the relative areas under the peaks in the elution profile, Fig. 1F) at 9:1 WLN5-6:hGrx1/Atox1. This was confirmed by separation of the proteins by anion exchange and colorimetric analyses (Fig. S2, where the hGrx1 and Atox1 proteins were resolved). These analyses indicate that on incubation, Cu transferred from Cu(I)-hGrx1 to the WLN5-6 protein preferentially (yielding Cu(I)-WLN5-6), despite the presence of equimolar Atox1 in the mixture. This result agrees with our observation that both the Atox1 and WLN5-6 *apo*-proteins can receive Cu from Cu(I)-hGrx1 (Figs. 1D, S2, S3).

Importantly, since Cu was not observed to transfer from Cu(I)-Atox1 to *apo-*hGrx1 or between Atox1 and WLN5-6, the predominance of Cu(I)-WLN5-6 as a product of this experiment, indicates direct, preferential Cu-transfer from Cu(I)-hGrx1 to *apo*-WLN5-6, rather than equilibration of Cu between the proteins in the mixture. That is, the distribution of Cu between the WLN5-6 and Atox1/Grx1 peaks cannot be accounted for by consideration of the Cu(I)-binding affinities of the Atox1 and WLN5-6 proteins alone, which indicate approximate two-fold tighter binding of Cu(I) to WLN5-6 *versus* Atox1 (Table S2). We therefore hypothesized that the Cu(I)-binding affinities of the individual proteins are not the only factors that determine Cu delivery from Cu(I)-hGrx1 when multiple protein partners are present and that the affinities of the protein-protein interactions between these proteins contribute to Cu transfer. We therefore sought to examine more closely the protein-protein interactions between the Cu(I)-hGrx1 and the Atox1 and WLN5-6 proteins.

### Confirmation that hGrx1 binds Cu(I) *via* two surface-exposed Cys residues

To confirm the location of the Cu(I) binding site in hGrx1 (between residues C23 and C26)^10^, we produced ^15^N and ^13^C/^15^N-labeled *apo*-hGrx1 and Cu(I)-hGrx1 for NMR studies. *Apo*-hGrx1 gave a high quality ^15^N-^1^H heteronuclear single quantum coherence (HSQC) spectrum with sharp and well dispersed peaks consistent with a well-folded and monomeric protein. Out of 115 peaks expected in the ^15^N-^1^H-HSQC spectrum from the 106-residue construct of *apo*-hGrx1 (excluding non-native residues from cleavage at the N-terminus), ~108 peaks were observed. Using standard triple resonance experiments, near complete HN-N, Cα and Cβ assignments of the *apo*-hGrx1 spectrum were made (96%, 95% and 95%, respectively), however peaks could not be assigned to residues T22, A50, T51, N52 and H53, suggesting this region undergoes conformational exchange (Fig. S4). In addition, Y25 and C26 presented as relatively weak peaks in most spectra. Conformational exchange for residues located at or near the C23-XX-C26 active site, was also reported in the NMR structural analysis of reduced hGrx1^45^. One peak in the *apo*-hGrx1 ^15^N-^1^H-HSQC spectrum (124.8/7.132 ppm) was not assigned as no corresponding signals could be observed in any of the triple resonance experiments. The assigned chemical shifts have been deposited into Biological Magnetic Resonance Bank (BMRB; accession number 27650).

A ^15^N-^1^H-HSQC spectrum of Cu(I)-hGrx1 was recorded and a comparison of the *apo*-hGrx1 and Cu(I)-hGrx1 spectra revealed that a subset of peaks (F18, I19, K20, C23, Y25, C26, I48, A67, T69, S84 and D85) showed significant chemical shift positional or signal intensity changes (Fig. S5A–C). These residues cluster in regions that encircle residues C23 and C26 (Fig. S5A–C), confirming these two cysteine residues are directly involved in the coordination of Cu(I). For example, the peak assigned to residue C23 showed one of the most significant positional changes, while the weak but observable peaks assigned to residues Y25 and C26 in the *apo*-hGrx1 spectrum disappeared completely in the Cu(I)-hGrx1 spectrum (Fig. S5A–C). In addition, the peak assigned to residue T69, located ~8Å from C23, shifted significantly and changed in its intensity. This residue has been demonstrated to be sensitive to the redox status and the structure of the active site including being displaced in structures minimized with the C23 thiolate group instead of a thiol^45,55^. Overall, this spectral comparison locates the Cu(I) binding site between residues C23 and C26, which agrees with previous mutagenesis and Cu binding studies of hGrx1^10^.

### NMR experiments reveal a role for protein-protein interactions in Cu transfer from Cu(I)-hGrx1 to WLN5-6

**Figure 2:**
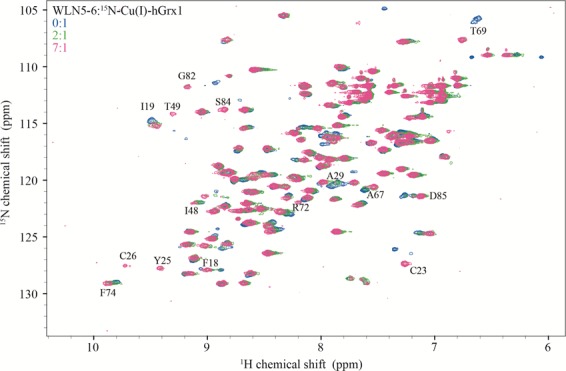
^15^N-^1^H-HSQC spectra of ^15^N-Cu(I)-hGrx1 titrated with WLN5-6. Overlay of ^15^N-^1^H-HSQC spectra of Cu(I)-hGrx1 before (blue) and after additions of WLN5-6 at Cu(I)-hGrx1:WLN5-6 molar ratios of 2:1 (green) and 7:1 (pink). Residues that display significant chemical shift changes (including positional and intensity) are labeled.

In our pairwise experiments, Cu(I)-hGrx1 was shown to transfer Cu to the *apo*-proteins Atox1 and WLN5-6 but the reverse transfers were not observed (Figs. S2, S3). To investigate the protein-protein interactions that occur during these unidirectional Cu(I)-exchange events, ^15^N-^1^H-HSQC titration studies were carried out by titrating unlabeled *apo*-proteins WLN5-6 or Atox1 into ^15^N-Cu(I)-hGrx1 to final molar ratios of 3.8:1 and 7.1:1 (partner:Cu(I)-hGrx1), respectively (Figs. 2, S6A,B, S7A–C). The changes in the ^15^N-^1^H-HSQC spectra indicated that Cu was displaced from Cu(I)-hGrx1 as increasing concentrations of partner proteins were added, with the ^15^N-^1^H-HSQC spectra largely resembling that of *apo*-hGrx1 at higher partner:Cu(I)-hGrx1 ratios (Figs. 2, S6A,B, S7A–C). These observations confirm Cu transfer from Cu(I)-hGrx1 to the *apo* partner proteins Atox1 and WLN5-6 as was observed by SEC-ICP-MS.

At higher concentrations of partner proteins, the observed spectral changes continued to progress, such that spectra recorded at partner:Cu(I)-hGrx1 ratios of 3.8:1 and 7.1:1 (partner:Cu(I)-hGrx1; for Atox1 and WLN5-6, respectively) showed additional spectral changes in comparison to the spectrum of *apo*-hGrx1. These observations may be ascribed to interactions between newly formed *apo*-hGrx1 and the partner proteins. That is, the formation of weak and transient *apo*-hGrx1-Cu(I)-Atox1 and *apo*-hGrx1-Cu(I)-WLN5-6 and/or *apo*-hGrx1-*apo*-Atox1 and *apo*-hGrx1-*apo*-WLN5-6 complexes. This indicates that *apo*-hGrx1-protein partner complexes persisted following Cu transfer. The spectral changes for these titrations, in terms of elucidating the molecular details and thermodynamics of these transient protein-protein complexes were challenging to decipher as they resulted from multiple hGrx1 species (*apo*-hGrx1, Cu(I)-hGrx1 and Cu(I)-hGrx1-partner, *apo*-hGrx1-Cu(I)-partner and *apo*-hGrx1-*apo*-partner protein complexes) that were present at varying concentrations throughout the titrations. Therefore, we conducted NMR titration studies with *apo*-^15^N-hGrx1 and the *apo*-proteins Atox1 and WLN5-6 in order to simplify the analyses and to directly measure the affinities of the protein-protein interactions.

### The same interaction surface facilitates Cu transfer from hGrx1 to Atox1 and WLN5-6

**Figure 3:**
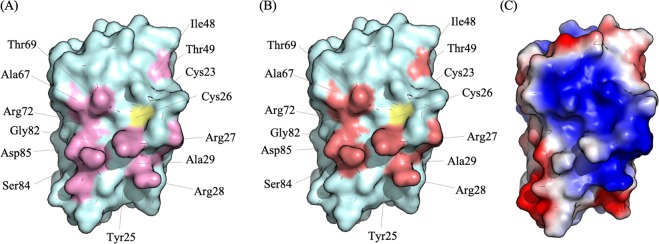
hGrx1 possess a common interaction site for the Atox1 and WLN5-6 proteins. (**A**) The hGrx1 interaction interface residues (labeled) with Atox1 are highlighted in pink on the hGrx1 surface representation (cyan). (**B**) The hGrx1 interaction interface residues (labeled) with WLN5-6 are highlighted in salmon on the hGrx1 surface representation (cyan). (**C**) The hGrx1 structure as represented in (**A,B**). The hGrx1 surface is colored according to the electrostatic potentials (red, negatively charged; blue, positively charged; white, uncharged). The active site Cys pair is marked in yellow. This figure was generated with PyMOL (The PyMOL Molecular Graphics System, Version 2.0 Schrödinger, LLC).

*Apo*-^15^N-hGrx1 was titrated with *apo*-proteins Atox1 or WLN5-6 to final molar ratios of 2.0:1 (partner:hGrx1) (Figs. S8A–C, S9A–C). In these titrations, variations were observed from the same set of peaks that showed changes at the highest partner:hGrx1 ratios in the Cu(I)-hGrx1 titrations. These included peaks assigned to residues at and surrounding the C23-XX-C26 Cu binding site, which map to a common region on the surface of hGrx1. This mapped region is consistent for both the Atox1 and WLN5-6 titrations and indicates that hGrx1 interacts with both protein partners using the same interaction surface (Fig. 3A,B). Calculation of the electrostatic surface potential of the hGrx1 structure shows that this interaction surface is predominantly positively charged (Fig. 3C). This aligns with previous studies of metallochaperone P_1B_-ATPase MBD interactions, which have reported that the complexes are mediated by complementary surface electrostatics^13,52,56^. The observed peak changes were fitted to a 1:1 binding model, which yielded *K*_D_s of 14 ± 6 μM and 7 ± 4 μM for the hGrx1-Atox1 and hGrx1-WLN5-6 interactions, respectively (Fig. S10A,B). These values are comparable to those determined for the protein-protein interactions between electron transfer partners^57^. Both metal and electron exchange require specific, yet transient protein-protein interactions. Given the same hGrx1 surface is involved in these protein-protein interactions, the difference in observed *K*_D_s for the hGrx1-Atox1 and hGrx1-WLN5-6 pairs is presumably due to differences in the surface structures of the Atox1 and WLN5-6 proteins (Fig. S11)^21,41,58,59^. Crucially, these observations indicate that protein-protein interactions between hGrx1 and the Atox1 and WLN5-6 proteins occur in the absence of Cu and that the affinities of these interactions are different between distinct pairs of protein partners.

## Conclusion

In this study, we established that hGrx1 binds Cu in a 1:1 stoichiometry at a C23-XX-C26 site and that hGrx1 can deliver Cu to the *apo-*proteins Atox1 and WLN5-6 in solution. Importantly, Cu transfer from hGrx1 to WLN5-6 occurs preferentially in the presence of Atox1 and the interactions of hGrx1 with the Atox1 and WLN5-6 are not Cu dependent.

The Cu-dependence of protein interactions that mediate intracellular Cu shuttling has been a controversial area of research. A number of studies have repeatedly stated that the interactions between proteins involved in Cu exchange are metal dependent. For example, examinations *via* NMR of Cu(I) delivery from Cu(I)-Atox1 to the multidomain protein ATP7B MBDs 1-6^34^, the determination of the structures of the Atx1-Ccc2a^52^ and complexes between Atox1 and the MBD1 of ATP7A from yeast and human, respectively^53^, all described the observed intermolecular adducts as metal-mediated. This is despite the fact that extensive intermolecular interfaces between the proteins in the complexes have been defined^52,53^, that the surface electrostatics of the proteins have been shown to be crucial for complex formation^53,56^ and that only minimal structural differences have been observed between *apo-* and Cu(I)-forms of these proteins and domains^37,60^. However, NMR experiments have also demonstrated interactions between MBDs 4–6 of ATP7B and Atox1 in the absence of Cu^27^. The results reported here give further support to the proposal that these interactions occur independently of the presence of Cu.

In a mixture of the Cu(I)-hGrx1, *apo-*Atox1, and *apo*-WLN5-6 proteins, Cu was preferentially delivered to the WLN5-6 protein, despite the fact that the *apo-*Atox1 and *apo*-WLN5-6 proteins showed only a two-fold difference in their respective *K*_DCu(I)_ values. This result can be reconciled by both the thermodynamics of the interactions between the proteins pairs and of the proteins with Cu(I). That is, the hGrx1 protein interacts with a higher affinity with the WLN5-6 protein than with the Atox1 and the binding affinity of WLN5-6 for Cu(I) is also higher. In fact, a calculation taking into account the respective values of *K*_DCu(I)_ for each protein, the *K*_D_s for the hGrx1-Atox1 and hGrx1-WLN5-6 interactions and the fact that the WLN5-6 protein binds two equivalents of Cu, predicts a Cu distribution for this experiment of approximately 0.6%, 87.6%, 11.8% for the hGrx1, WLN5-6 and Atox1 proteins respectively, which correlates with our analysis. Interestingly, both proteins (Atox1 and WLN5-6) interact at the same interface with hGrx1, which is proximate to the Cu(I)-binding site. The predicted consequence of the different affinities of these proteins for hGrx1 is competition for binding at this common site and therefore competition for Cu exchange. Importantly, the outcome of this ‘competition’ in the cell, would not only be determined by the relative affinities of each protein for Cu(I) and for each other, but also the abundances of each protein^61^.

Crucially, we show here that hGrx1 is able to transfer Cu to the WLN5-6 protein, even in the presence of Atox1. To date, Atox1 has been the sole metallochaperone protein proposed to act in Cu delivery to the ATP7A and ATP7B proteins. The fact that hGrx1 can act in this way is significant for intracellular Cu homeostasis. This finding suggests a potential redundancy in this system and a role for hGrx1 under cellular conditions where the activity of Atox1 in Cu regulation is attenuated. For example, a number of lines of evidence suggest that both the cellular ratios of *apo*-Atox1/Cu(I)-Atox1 and reduced/oxidized Atox1 influence metabolic Cu flux and specifically the activity and trafficking of the Cu ATPases^37,38,62^. There is also evidence to suggest that Atox1 is not absolutely required for Cu delivery to the Cu(I)-ATPases^59,63^ and that other Cu carriers may supplement Atox1 function. For example, *ATOX1* knockout does not completely abrogate ATP7A function, suggesting that ATP7A may obtain Cu from alternative metal donor(s)^59,63^. The data presented in this study suggests that hGrx1 could assume such a role.

## Methods

### Protein overexpression and purification

The DNA sequence encoding hGrx1 was amplified *via* PCR and subcloned into a pGEX-6P-1 glutathione S-transferase fusion vector with an intervening PreScission Protease site for cleavage of the GST tag. The DNA sequences encoding Atox1 and the WLN5-6 protein fragment (encoding residues 486–633 of ATP7B) were amplified *via* PCR and subcloned into the pTEM-11 and pET-24d vectors, respectively. The pGEX-6p-1-hGrx1, pTEM-11-Atox1 and pET-24d-WLN5-6 plasmids were individually transformed into *Escherichia coli* strain BL21 Codon Plus (DE3). Cultures were grown in Luria Broth (LB) at 37 °C to an optical density OD_600_ of approximately 0.8, induced with isopropyl β-D-1-thiogalactopyranoside (IPTG, 0.5 mM) and harvested after 16 h with shaking at 25 °C. The hGrx1 isotope labeled samples (^15^N and/or ^13^C) were grown in LB at 37 °C to an OD_600_ of 0.6–0.8, harvested and washed twice in M9 salts before transfer to labeled M9 media and induction with IPTG (1.0 mM). Cells were harvested by centrifugation after 16 h with shaking at 25 °C^64^.

The hGrx1 and ^15^N-^13^C-labeled hGrx1 proteins were purified by glutathione S-transferase (GST) affinity chromatography^65,66^. Frozen cell pellets were thawed at room temperature and resuspended in cell lysis buffer (phosphate-buffered saline (PBS), 1 mM Tris (2-carboxyethyl) phosphine hydrochloride (TCEP)). Cells were disrupted by passage through a TS series bench top cell disruptor (Constant Systems Ltd) at 35 kpsi. Cell debris was removed by centrifugation (Beckman JLA-25.50, 30000 g, 20 min, 4 °C) and the soluble fraction was incubated with glutathione sepharose 4B resin (GE Healthcare) equilibrated with lysis buffer. The GST tag was cleaved with PreScission Protease (GE Healthcare) followed by size-exclusion chromatography (SEC; HiLoad 16/600 Superdex 75 pg, GE Healthcare; 20 mM Tris-MES, 150 mM NaCl, 1 mM TCEP, pH 8.0). Purified proteins were concentrated to 20 mg/mL before storage at −80 °C.

The purifications of the Atox1 and WLN5-6 proteins were conducted according to previously reported protocols with minor modifications^21,44^. The Atox1 was purified by cation exchange chromatography (HiTrap SP FF, GE Healthcare), eluted with a linear NaCl gradient (0.0–1.0 M, 20 mM sodium acetate, 1 mM TCEP, pH 5.0), followed by SEC (HiLoad 16/600 Superdex 75 pg, GE Healthcare; 20 mM potassium phosphate, 150 mM NaCl, 1 mM TCEP, pH 7.0). The WLN5-6 protein was isolated by anion exchange chromatography (HiTrap Q FF, GE Healthcare), and was eluted with a linear NaCl gradient (0.0–1.0 M in 20 mM sodium MES 0.1 mM EDTA, 1 mM TCEP, pH 6.0), followed by SEC (HiLoad 16/600 Superdex 75 pg, GE Healthcare; 20 mM MES/Na, 150 mM NaCl, 1 mM TCEP, pH 5.5). Purified proteins were concentrated to 20 mg/mL prior to storage at −80 °C.

### Cu-loading

The purified proteins were exchanged into buffer (20 mM Tris-MES, pH 8.0) by centrifugal ultrafiltration (Millipore) and incubated for 30 mins with CuSO_4_ and reduced GSH (molar ratio 1:5:10; protein: CuSO_4_: GSH). In order to remove the excess Cu from the mixture, the incubated protein sample was applied to a SEC column (HiLoad 16/600 Superdex 75 pg, GE Healthcare). The presence of Cu(I) in the peak fractions was analyzed colorimetrically using the ligand bathocuproinedisulfonic acid (Bcs) and those protein fractions containing Cu were pooled and concentrated by centrifugal ultrafiltration. These analyses indicated excellent resolution between the Cu(I)-protein and excess Cu-GSH peaks (data not shown). The Cu(I):protein stoichiometries of the protein samples prepared in this manner were confirmed by a colorimetric assay using Bcs^10^.

### Measurement of Cu-binding

The Cu(I) probe ligand Bcs, which reacts quantitatively with Cu(I) to yield a 1:2 complex [Cu^I^Bcs_2_]^3−^ with a characteristic solution spectrum (ϵ = 13000 M^−1^cm^−1^ at λ_max_ 483 nm) and a defined formation constant (logβ_2_ = 19.8)^10^ was employed for the quantification of Cu(I) binding to all proteins^10^. Briefly, purified proteins (20 mM Tris-MES, pH 7.0) were titrated at various concentrations (1–30 μM) into solutions containing buffer (20 mM Tris-MES, pH 7.0), CuSO_4_ (20 μM), Bcs (200 μM) and reduced GSH (400 μM). The exchange of Cu(I) from the [Cu^I^Bcs_2_]^3−^ complex to the proteins was monitored by measuring the absorbance of the resulting solutions at 483 nm. The data were analyzed as previously described^10,67^.

### Cu-exchange

Protein samples (2 μg) of Cu(I)-loaded, *apo*-proteins and protein mixtures at 1:1 molar ratios were applied to a Bio-SEC. 3 column (3 mm particle size; 150 Å pore structure; 4.6 mm i.d., Agilent Technologies) in 200 mM ammonium nitrate (pH 7.6−7.8, adjusted with ammonium hydroxide) with 10 μg/L cesium (Cs) and antimony (Sb) as internal standards. Chromatographic separations of the proteins were monitored by measuring the UV absorbance of the eluent at 280 nm (indicating the presence of protein) and inductively coupled plasma mass spectrometry (ICP-MS) (Agilent Technologies 7700 x ICP-MS)^68^ was used to monitor the presence of Cu.

For the analyses of protein mixtures that contained the hGrx1 and Atox1 proteins (which could not be resolved by SEC), separation of the proteins by anion exchange was carried out. Protein samples were applied to a mono Q 5/50 GL column (GE Healthcare) pre-equilibrated with binding buffer (20 mM Tris-MES, pH 8.0) and bound proteins were eluted by the application of a linear NaCl gradient (0.0–1.0 M NaCl, 20 mM Tris-MES, pH 8.0). The Cu contents of the eluted fractions were determined colorimetrically with Bcs^10^.

### NMR spectroscopy

^15^N-^1^H heteronuclear single quantum coherence (HSQC) spectra were recorded at 4 °C using a Bruker Avance III 600 MHz NMR spectrometer equipped with a triple-resonance TCI cryogenic probehead. All stock protein solutions were dialyzed against MES/Na buffer (10 mM MES/Na, 50 mM NaCl, 1 mM TCEP, pH 6.0) overnight prior to titration studies. ^15^N-^1^H-HSQC spectra of ^15^N-*apo* and ^15^N-Cu(I)-hGrx1 (350–400 μl at 320–350 μM) were recorded after sequential additions of unlabeled *apo* forms of the proteins Atox1 or WLN5-6. For the titrations into ^15^N-*apo*-hGrx1, the *apo*-proteins Atox1 or WLN5-6 (0.46 and 0.5 mM, respectively) were titrated to final molar ratios of 2.0:1 (partner:apo hGrx1). For titrations into ^15^N-Cu(I)-hGrx1, final molar ratios of 3.8:1 and 7.1:1 (partner:hGrx1) were obtained upon sequential additions of the *apo*-proteins Atox1 or WLN5-6 (0.5 and 1.5 mM, respectively). Higher molar ratios lead to the observation of some protein precipitation and deterioration in the quality of the spectra recorded. D_2_O was added to each sample to a final concentration of 5% (v/v). Spectra were processed with Topspin 3.5 (Bruker Biospin) and analyzed using SPARKY (T. D. Goddard and D. G. Kneller, University of California, San Francisco). Spectral changes (either in intensity or peak position) were plotted against varying concentrations of the titrants. For peak positional changes, combined chemical shift perturbations, Δ chemical shift (ppm) were calculated based on the equation Δδ_ppm_ = [(Δδ_HN_)^2^ + (0.154 × Δδ_N_)^2^]^1/2^ where Δδ_HN_ and Δδ_N_ represent the chemical shift variations in the proton and nitrogen dimensions, respectively. Comparing *apo* and Cu(I)-hGrx1 spectra, peaks that were not overlapped and with positions changing by more than one standard deviation (SD) over the mean or those with intensity decreasing by >75% or increasing by >100% were deemed to be significantly affected. For titration with partner proteins, peaks with positions changed by more than one standard deviation (SD) over the mean or those with intensity changes >50% at molar ratios beyond 1:1 were deemed to be significantly affected. Resonances corresponding to a number of residues were initially evaluated. The peaks corresponding to Cys26 and Thr69 showed the clearest and most significant changes amongst those analyzed and therefore these were used for the affinity calculations. Binding affinities were determined from non-linear-least-square curve fitting to a 1:1 binding model based on intensity changes using Origin2016.

### NMR assignment of *apo*-hGrx1

hGrx1 assignments were unavailable from the previously determined *apo*-hGrx1 structure (PDB code (1JHB)^45^), so triple resonance experiments were recorded on a purified ^13^C/^15^N-labeled hGrx1 sample (0.5 mM) to allow the assignment of the ^15^N-^1^H-HSQC spectrum. Spectra were analyzed with Sparky (T. D. Goddard and D. G. Kneller, University of California, San Francisco). Backbone ^15^N, H^N^, ^13^C and ^13^C resonances were obtained from HNCACB, CBCA(CO)NH, HCC(CO)NH and CC(CO)NH experiments using standard methods. ^15^N and ^13^C chemical shifts were referenced indirectly using 4,4-dimethyl-4-silapentane-1-sulfonic acid (DSS) according to their magnetogyric ratios.

## Supporting Information

Supporting Information.
