# Supplementary material for: Human glutaredoxin-1 can transfer copper to isolated metal binding domains of the P1B-type ATPase, ATP7B
Source: Sci Rep. 2020 Mar 5;10:4157. doi: 10.1038/s41598-020-60953-z (PMC7057996; doi:10.1038/s41598-020-60953-z)
Supplement: Supplementary file 1 — Supporting Information. [file 41598_2020_60953_MOESM1_ESM.pdf]

SUPPLEMENTARY INFORMATION

Human glutaredoxin-1 can transfer copper to isolated metal binding domains of the P_{1B}-type ATPase, ATP7B

Shadi Maghool[1], Sharon La Fontaine[2,3], Blaine R. Roberts[3], Ann H. Kwan[4,*] and Megan J. Maher[1,5*]

[1]*Department of Biochemistry and Genetics, La Trobe Institute for Molecular Science, La Trobe University, Melbourne, VIC Australia.*

[2]*School of Life and Environmental Sciences, Deakin University, Geelong, VIC Australia.*

[3]*The Florey Institute of Neuroscience, The University of Melbourne, Parkville, VIC, Australia.*

[4]*School of Life and Environmental Sciences, The University of Sydney, Sydney, NSW Australia.*

[5]*School of Chemistry and The Bio21 Molecular Science and Biotechnology Institute, The University of Melbourne, Parkville, Australia.*

[*]Corresponding authors:

A/Prof. Megan Maher,
School of Chemistry and The Bio21 Molecular Science and Biotechnology Institute,
The University of Melbourne,
Melbourne, Victoria, 3010 Australia.
Phone: +61 3 9035 7451
E-mail: megan.maher@unimelb.edu.au

Dr Ann Kwan,
School of Life and Environmental Sciences and Sydney Nano Institute,
University of Sydney,
Maze Crescent,
Sydney, NSW 2006, Australia
Phone: +61 2 9351 3911
E-mail: ann.kwan@sydney.edu.au

Table S1 Cu:protein stoichiometries for purified proteins

Protein	[Cu]/[Protein]
hGrx1	0.97 ± 0.02
Atox1	1.01 ± 0.05
WLN5-6	1.94 ± 0.03

Table S2 Apparent Cu(I) dissociation constants K_D for the Cu(I)-binding sites for the hGrx1, Atox1 and WLN5-6 proteins, determined *via* competition with Bcs.

Protein	Log K_D pH 7.0	
	This work	Ref
hGrx1	-15.8	-15.5[10]
Atox1	-17.5	-17.4[10,44]
WLN5-6	-17.8	-17.6[44]

Supplementary Figure Captions

Figure S1 Analyses of purified ^{15}N-hGrx1, Atox1, hGrx1 and WLN5-6 proteins by Coomassie Brilliant Blue (CBB)-stained SDS-PAGE. Pre-stained SDS-PAGE protein marker (Bio-Rad, lane 1) and protein samples (10 μg, lanes 2-5) were resolved by electrophoresis with a 10% Bis-Tris gel under reducing conditions (Thermo Fisher Scientific).

Figure S2

Results of Cu exchange reactions between Cu(I)-hGrx1 and partner proteins. (A) Cu(I)-hGrx1, (B) Cu(I)-Atox1 and (C) Cu(I)-WLN5-6 were applied to an anion exchange column and fractions analyzed for the presence of protein (A_{280}, solid lines) and for Cu(I) with Bcs (the $[Cu^{I}Bcs_2]^{3-}$ complex is detected colorimetrically at A_{483}, dashed orange lines). (D) Results of Cu exchange reaction between Cu(I)-hGrx1 and *apo*-Atox1. Cu(I)-hGrx1 and *apo*-Atox1 were incubated together at 1:1 molar and re-separated using anion exchange. (E) Cu exchange between Cu(I)-Atox1 and *apo*-hGrx1. (F) Cu exchange between Cu(I)-WLN5-6 and *apo*-Atox1 (G) Cu exchange between Cu(I)-Atox1 and *apo*-WLN5-6. (H) Cu exchange between Cu(I)-hGrx1, and a mixture of the *apo*-Atox1 and *apo*-WLN5-6 proteins. Cu(I)-hGrx1, *apo*-Atox1 and *apo*-WLN5-6 were incubated together at molar ratio of 1:1:1 and re-separated using anion exchange (A_{280}, pink solid line) and fractions colorimetrically analysed for the presence of Cu (orange dashed line).

Figure S3 Schematic representations of related Cu exchange experiments conducted in this work. The ✔ symbol indicates that Cu transferred between proteins and the ✗ symbol indicates that it is not. This figure was generated with Microsoft PowerPoint for Mac (Version 16.31).

Figure S4 Assigned ^{15}N-^{1}H-HSQC spectrum of ^{15}N-^{13}C *apo*-hGrx1. Lines indicate sidechain resonances from Asn and Gln residues.

Figure S5A Comparison of ^{15}N-^{1}H-HSQC spectra of *apo*-hGrx1 and Cu(I)-hGrx1. (A) Overlay of ^{15}N-^{1}H-HSQC spectra of *apo*-hGrx1(red) and Cu(I)-hGrx1(blue). Residues that display significant chemical shift changes (including positional and intensity) are labeled. Arrows show groups of peaks that belong to the same amino acid in the two states.

Figure S5B Histogram showing combined H and N chemical shift changes comparing ^{15}N-Cu(I)-hGrx1 and ^{15}N-*apo*-hGrx1.

Figure S5C Histogram showing changes in peak heights comparing ^{15}N-Cu(I)-hGrx1 and ^{15}N-*apo*-hGrx1.

Figure S6A Histogram showing combined H and N chemical shift changes during WLN5-6 titrations to ^{15}N-Cu(I)-hGrx1. Results were shown for titrations of WLN5-6 to ^{15}N-Cu(I)-hGrx1 at molar ratios of 1:1 (blue), 2:1 (green) and 7:1 (pink).
Figure S6B Histogram showing changes in peak heights during WLN5-6 titrations during WLN5-6 titrations to ^{15}N-Cu(I)-hGrx1. Results were shown for titrations of WLN5-6 to ^{15}N-Cu(I)-hGrx1 at molar ratios of 1:1 (blue), 2:1 (green) and 7:1 (pink).

Figure S7A ^{15}N-^{1}H-HSQC spectra of ^{15}N-Cu(I)-hGrx1 titrated with Atox1. Overlay of ^{15}N-^{1}H-HSQC spectra of Cu(I)-hGrx1 before (blue) and after additions of Atox1 at Cu(I)-hGrx1:Atox1 molar ratios of 2.2:1 (green) and 3.8:1 (pink). Residues that display significant chemical shift changes (including positional and intensity) are labelled.
Figure S7B Histogram showing combined H and N chemical shift changes during Atox1 titrations to ^{15}N-Cu(I)-hGrx1. Results were shown for titrations of Atox1 to ^{15}N-Cu(I)-hGrx1 at molar ratios of 2.2:1 (blue) and 3.8:1 (pink).
Figure S7C Histogram showing changes in peak heights during Atox1 titrations during Atox1 titrations to ^{15}N-Cu(I)-hGrx1. Results were shown for titrations of Atox1 to ^{15}N-Cu(I)-hGrx1 at molar ratios of 2.2:1 (blue) and 3.8:1 (pink).

Figure S8A. ^{15}N-^{1}H-HSQC spectra of ^{15}N-*apo*-hGrx1 titrated with Atox1. Overlay of ^{15}N-^{1}H-HSQC spectra of *apo*-hGrx1 before (red) and after additions of Atox1 at *apo*-hGrx1:Atox1 molar ratios of 0.1:1 (black) and 1.1:1 (cyan). Residues that display significant chemical shift changes (including positional and intensity) are labeled.
Figure S8B Histogram showing combined H and N chemical shift changes during Atox1 titrations to ^{15}N-*apo*-hGrx1. Results were shown for titrations of Atox1 to ^{15}N-*apo*-hGrx1 at molar ratios of 0.1:1 (black) and 1.1:1 (cyan).
Figure S8C Histogram showing changes in peak heights during Atox1 titrations during Atox1 titrations to ^{15}N-*apo*-hGrx1. Results were shown for titrations of Atox1 to ^{15}N-*apo*-hGrx1 at molar ratios of 0.1:1 (black) and 1.1:1 (cyan).

Figure S9A ^{15}N-^{1}H-HSQC spectra of ^{15}N-*apo*-hGrx1 titrated with WLN5-6. Overlay of ^{15}N-^{1}H-HSQC spectra of *apo*-hGrx1 before (red) and after additions of WLN5-6 at *apo*-hGrx1:WLN5-6 molar ratios of 0.2:1 (black) and 1:1 (cyan). Residues that display significant chemical shift changes (including positional and intensity) are labeled.

Figure S9B Histogram showing combined H and N chemical shift changes during WLN5-6 titrations to ^{15}N-*apo*-hGrx1. Results were shown for titrations of WLN5-6 to ^{15}N-*apo*-hGrx1 at molar ratios of 0.2:1 (black) and 1:1 (cyan).

Figure S9C Histogram showing changes in peak heights during WLN5-6 titrations during WLN5-6 titrations to ^{15}N-*apo*-hGrx1. Results were shown for titrations of WLN5-6 to ^{15}N-*apo*-hGrx1 at molar ratios of 0.2:1 (black) and 1:1 (cyan).

Figure S10A ^{15}N-^{1}H-HSQC titrations of Atox1 into ^{15}N-*apo*-hGrx1 in absence of Cu(I). Peak heights (arbitrary units, black squares) and fitted values (lines) for a 1:1 binding model are shown for residues C23 and T69. Residuals of the fits are shown as red squares in the bottom plots.

Figure S10B ^{15}N-^{1}H-HSQC titrations of WLN5-6 into ^{15}N-*apo*-hGrx1 in absence of Cu(I). Peak heights (arbitrary units, black squares) and fitted values (lines) for a 1:1 binding model are shown for residues C23 and T69. Residuals of the fits are shown as red squares in the bottom plots.

Figure S11 Electrostatic surface structures of hGrx1, Atox1 and WLN5-6. (A) hGrx1 (PDB code 4RQR[41]), (B) Atox1 (PDB code 1FEE[58]), (C) WLN5-6 (PDB code 2EW9[21]). The top panels show the electrostatic surface structures of the proteins, colored according to the electrostatic potentials (red: negatively charged; blue: positively charged; white: uncharged). In the bottom panels the secondary structures of the proteins are represented as cartoons in identical orientations. hGrx1: cyan; Atox1: pink and WLN 5-6: gray. Cysteine residues in the C-XX-C motifs are shown as yellow spheres. This figure was generated with PyMOL (The PyMOL Molecular Graphics System, Version 2.0 Schrödinger, LLC).

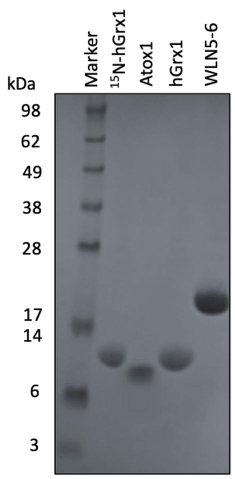

Figure S1.

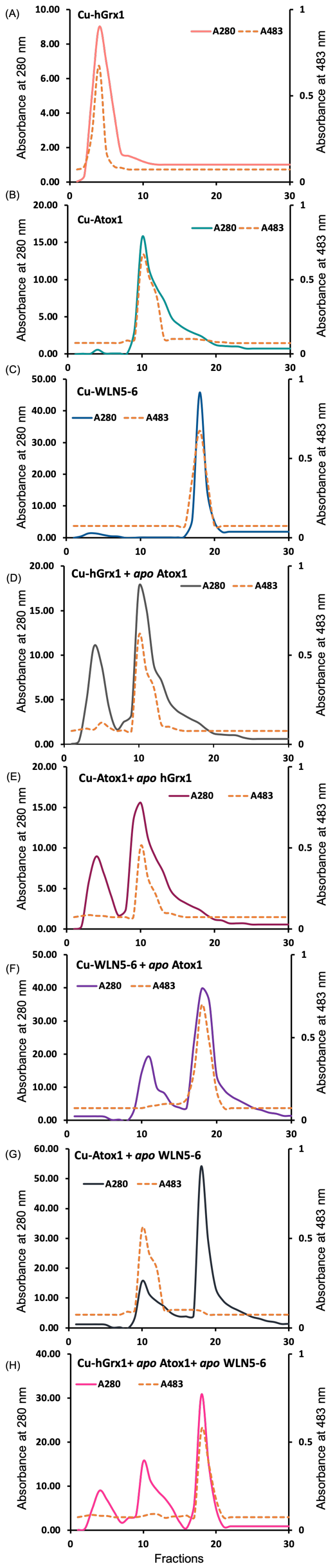

156

157 **Figure S2.**

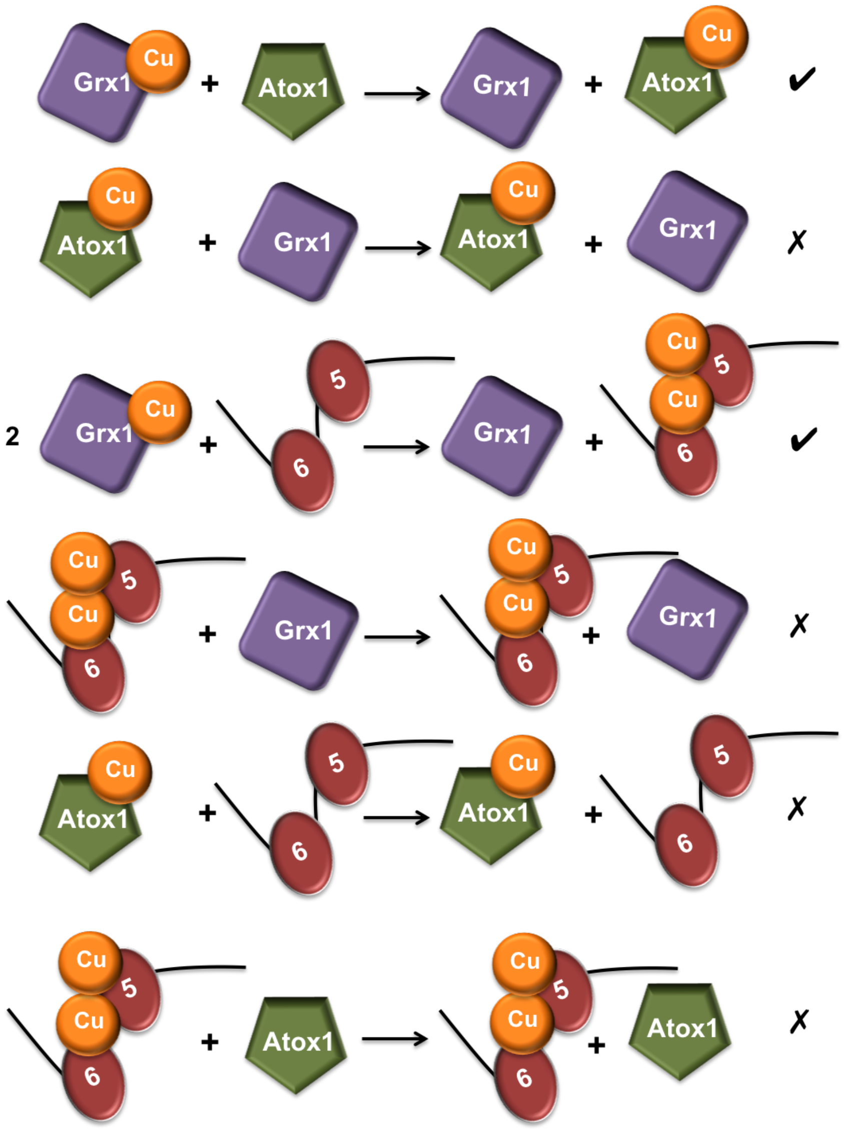

Figure S3.

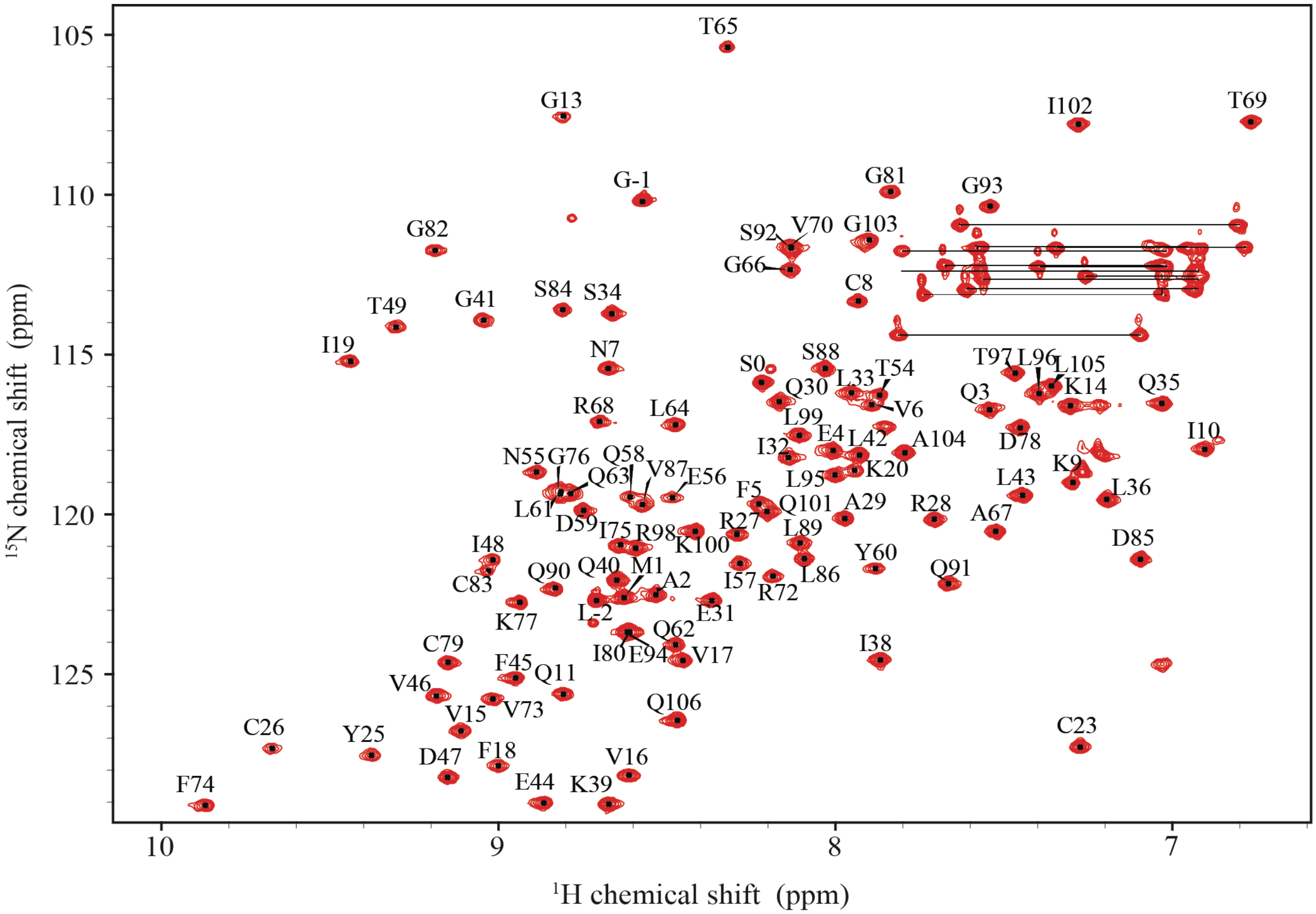
T65
G13
I102
T69
G-1
G81
G93
G82
S92
V70
G103
G66
C8
T49
G41
S84
S34
I19
N7
S0
S88
L33
T54
T97
L96
L105
Q30
V6
K14
Q35
R68
L64
Q3
L99
I32
E4
L42
A104
D78
I10
N55
G76
Q58
Q63
V87
E56
K20
K9
L95
L43
F5
L36
L61
Q101
A29
D59
R28
A67
R27
I75
R98
L89
K100
D85
I48
Y60
Q90
Q40
M1
L86
Q91
C83
A2
I57
R72
K77
E31
L-2
Q62
I38
C79
I80
E94
V17
F45
Q11
V46
V73
V15
Q106
C26
C23
Y25
D47
F18
V16
F74
E44
K39
105
110
115
120
125
10
9
8
7
^{15}N chemical shift (ppm)
^{1}H chemical shift (ppm)

Figure S4.

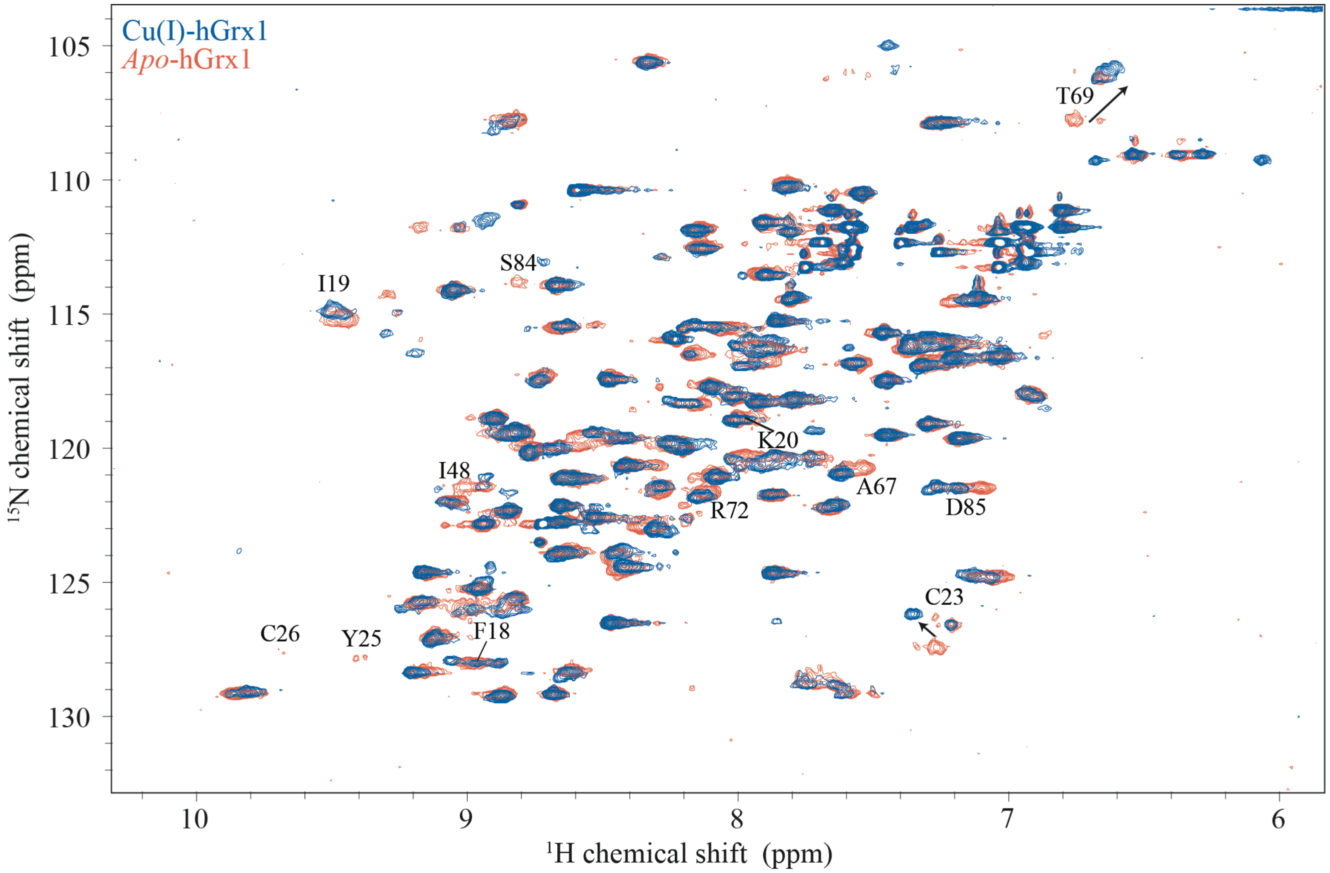
Cu(I)-hGrx1
Apo-hGrx1
T69
S84
I19
K20
I48
A67
R72
D85
C23
C26
Y25
F18
105
110
115
120
125
130
^{15}N chemical shift (ppm)
10
9
8
7
6
^{1}H chemical shift (ppm)

Figure S5A

Combined Δ_{H+N} chemical shift comparing Cu(I)-hGrx1 to *Apo*-hGrx1

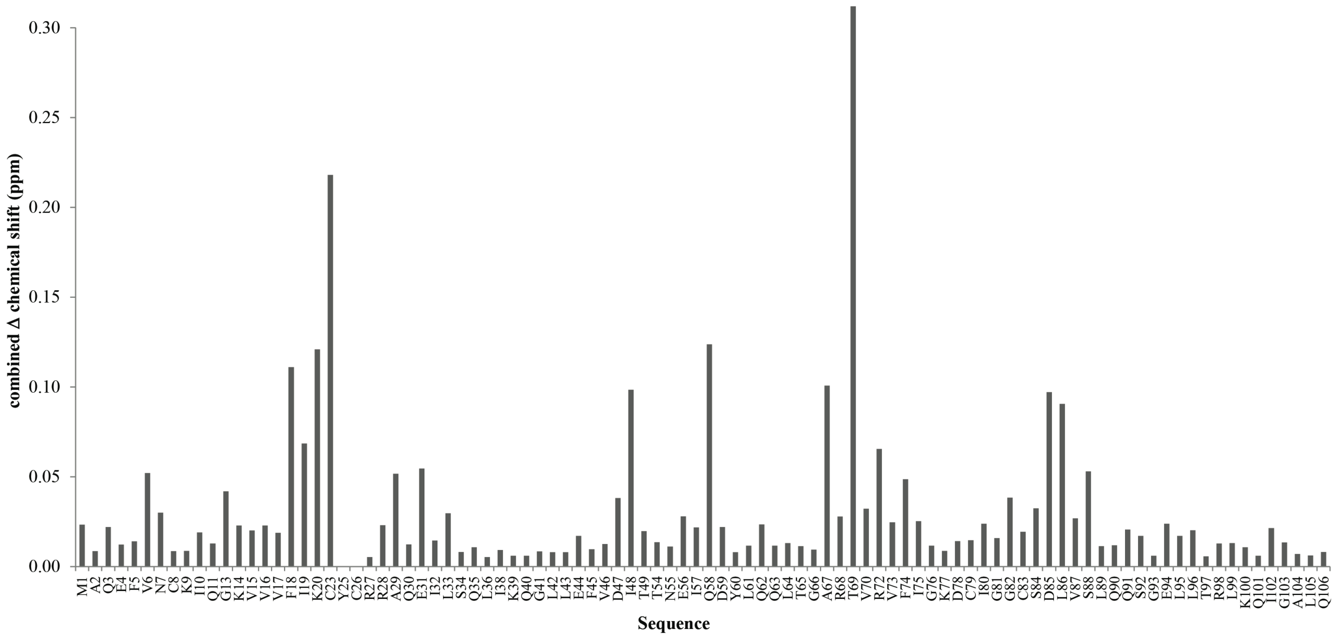

Figure S5B

Scaled peak intensity of Cu(I)-hGrx1 vs *Apo*-hGrx1

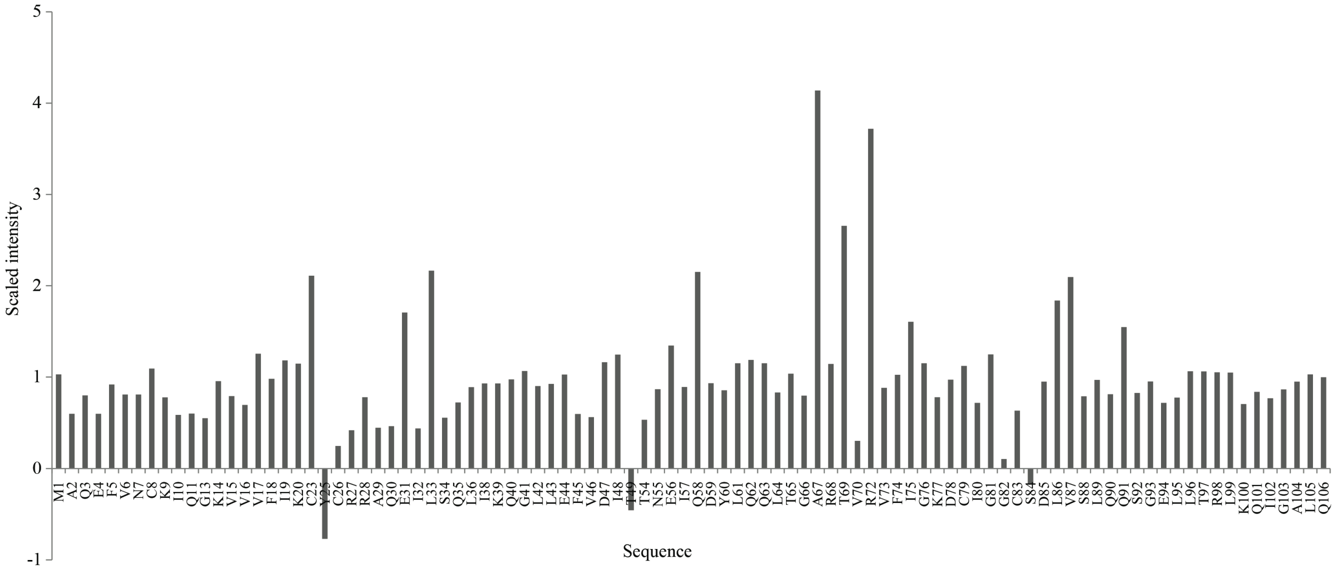

Figure S5C

Combined Δ_{H+N} chemical shift of ^{15}N-Cu(I)-hGrx1 titrated with WLN5-6

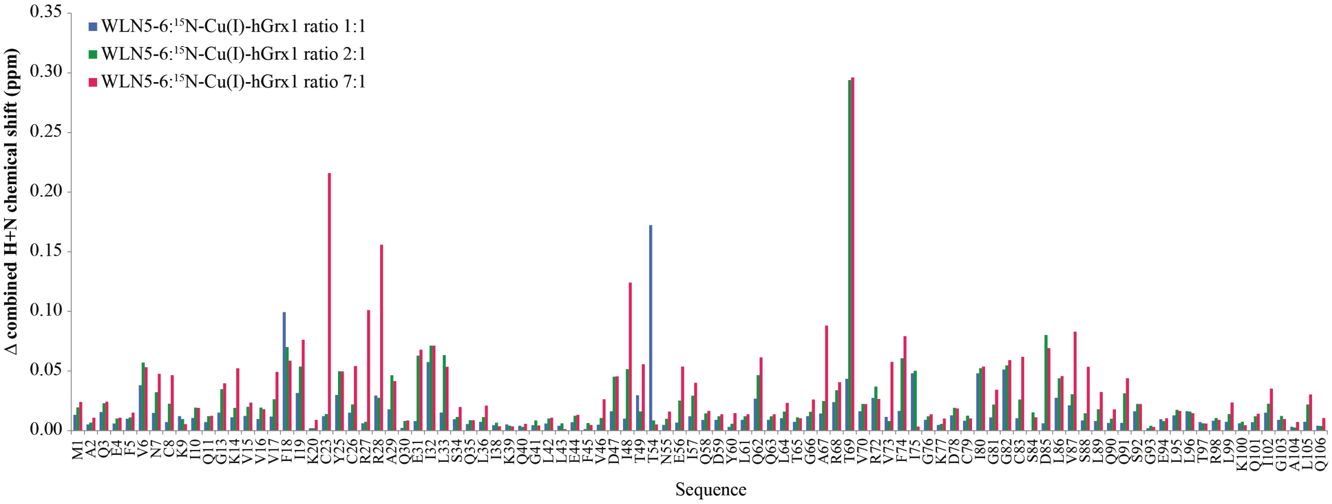

Figure S6A.

Scaled peak intensity of ^{15}N-Cu(I)-hGrx1 titrated with WLN5-6

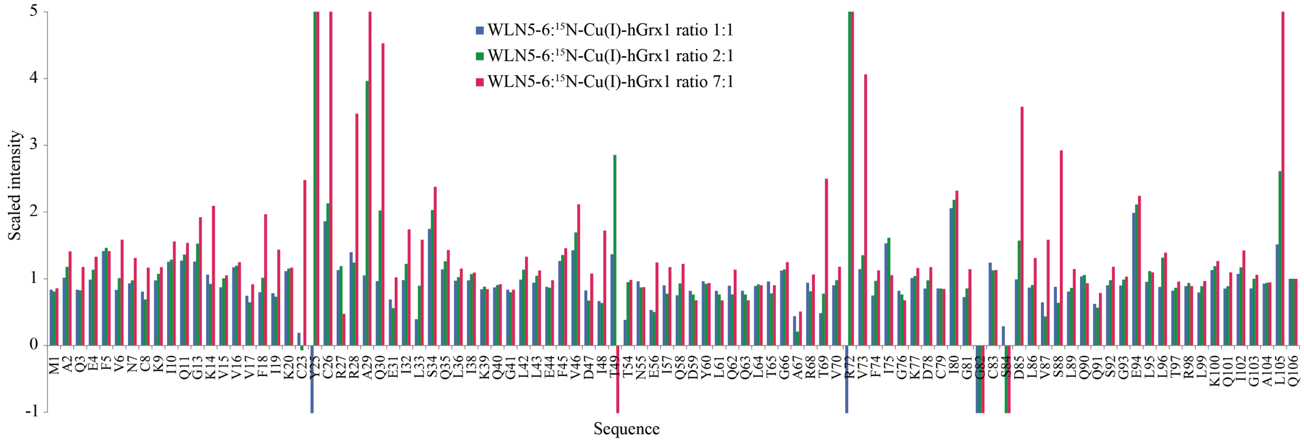

Figure S6B.

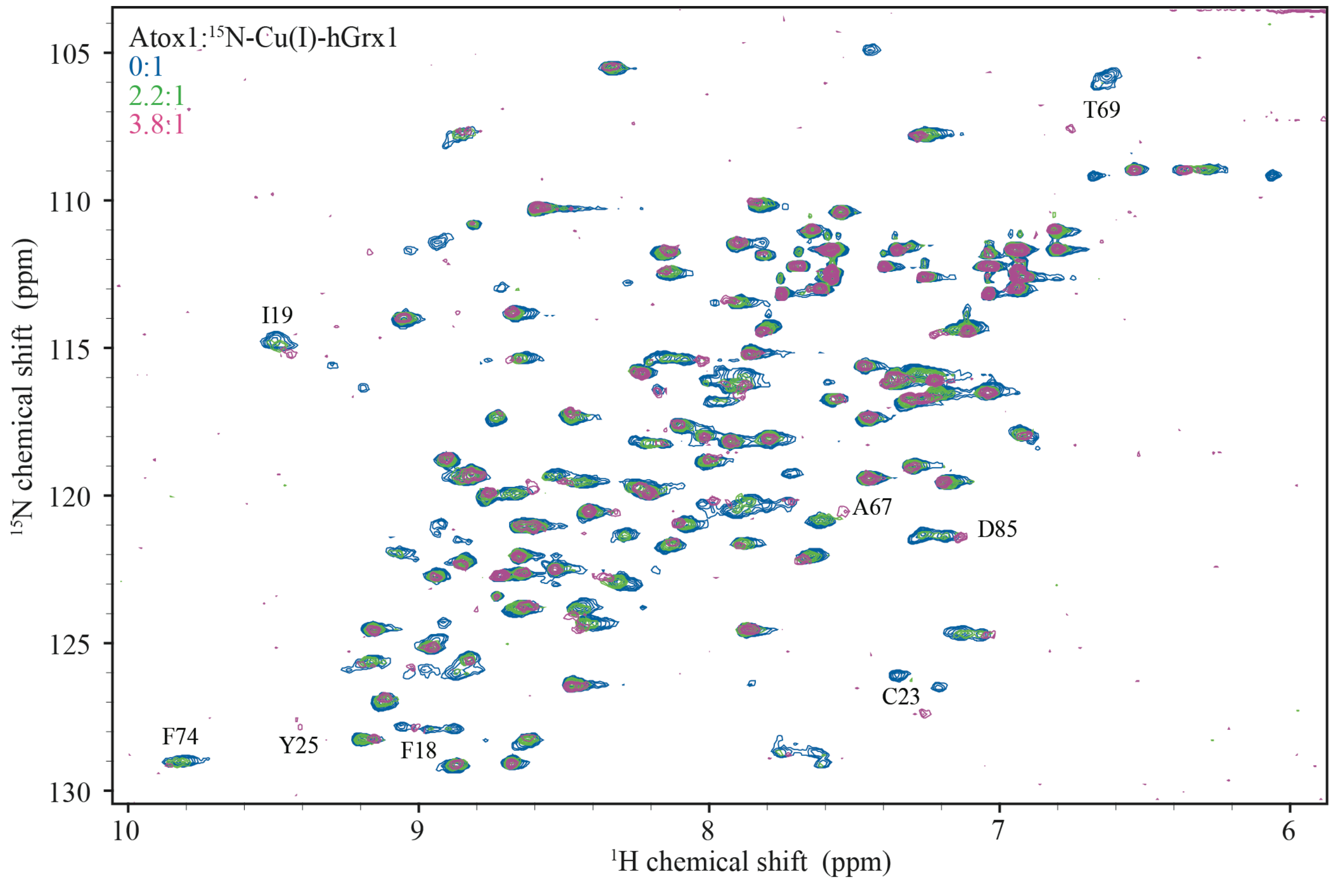
Atox1:15N-Cu(I)-hGrx1
0:1
2.2:1
3.8:1
T69
I19
A67
D85
C23
F74
Y25
F18
15N chemical shift (ppm)
1H chemical shift (ppm)
105
110
115
120
125
130
10
9
8
7
6

Figure S7A.

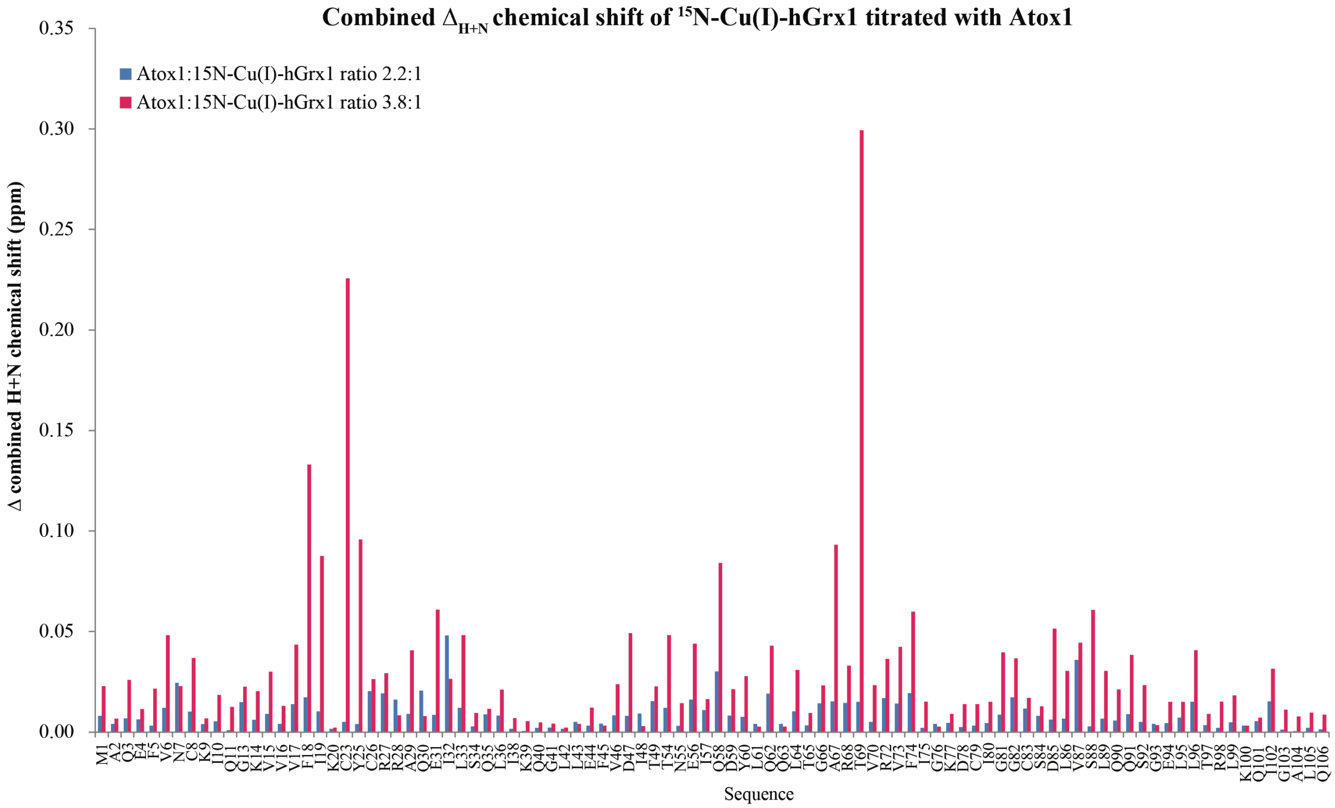

Figure S7B.

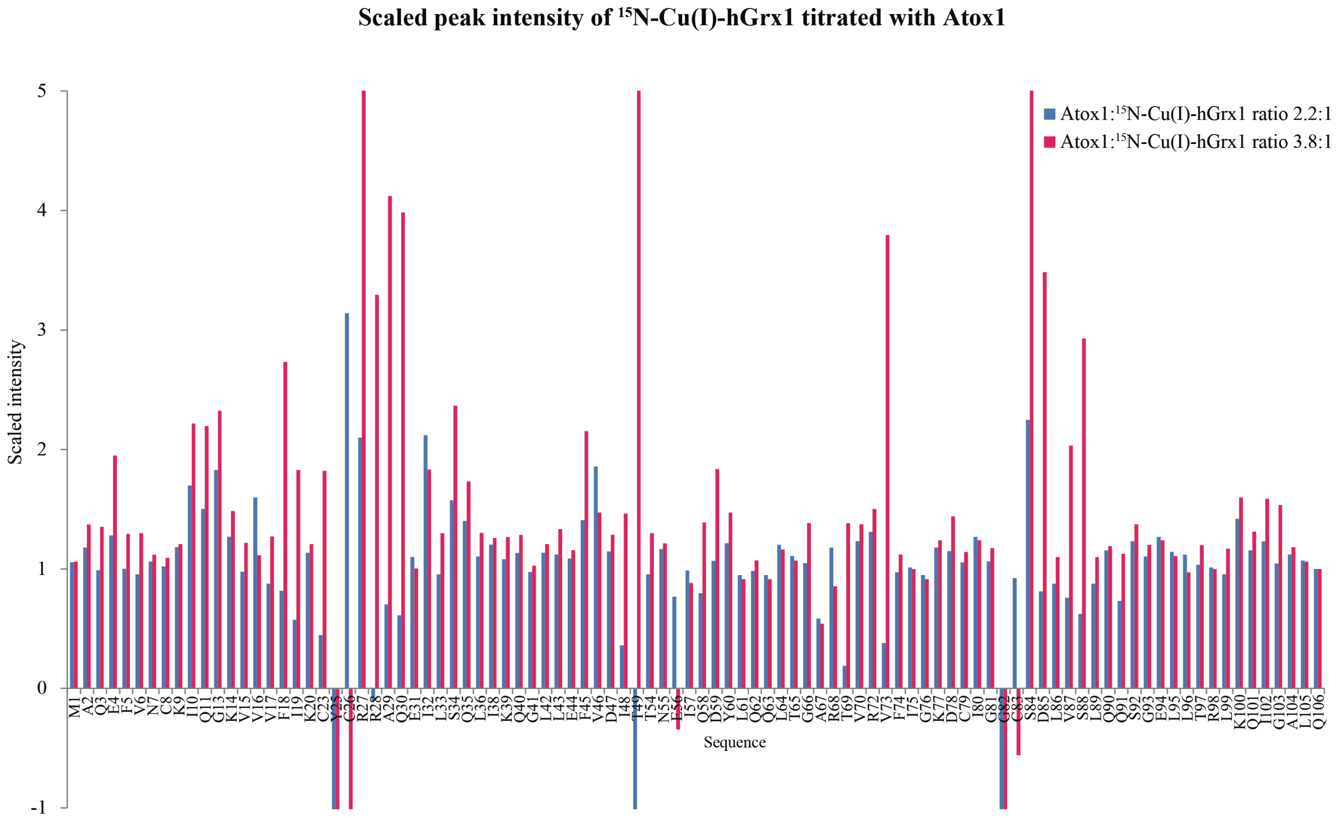

Figure S7C.

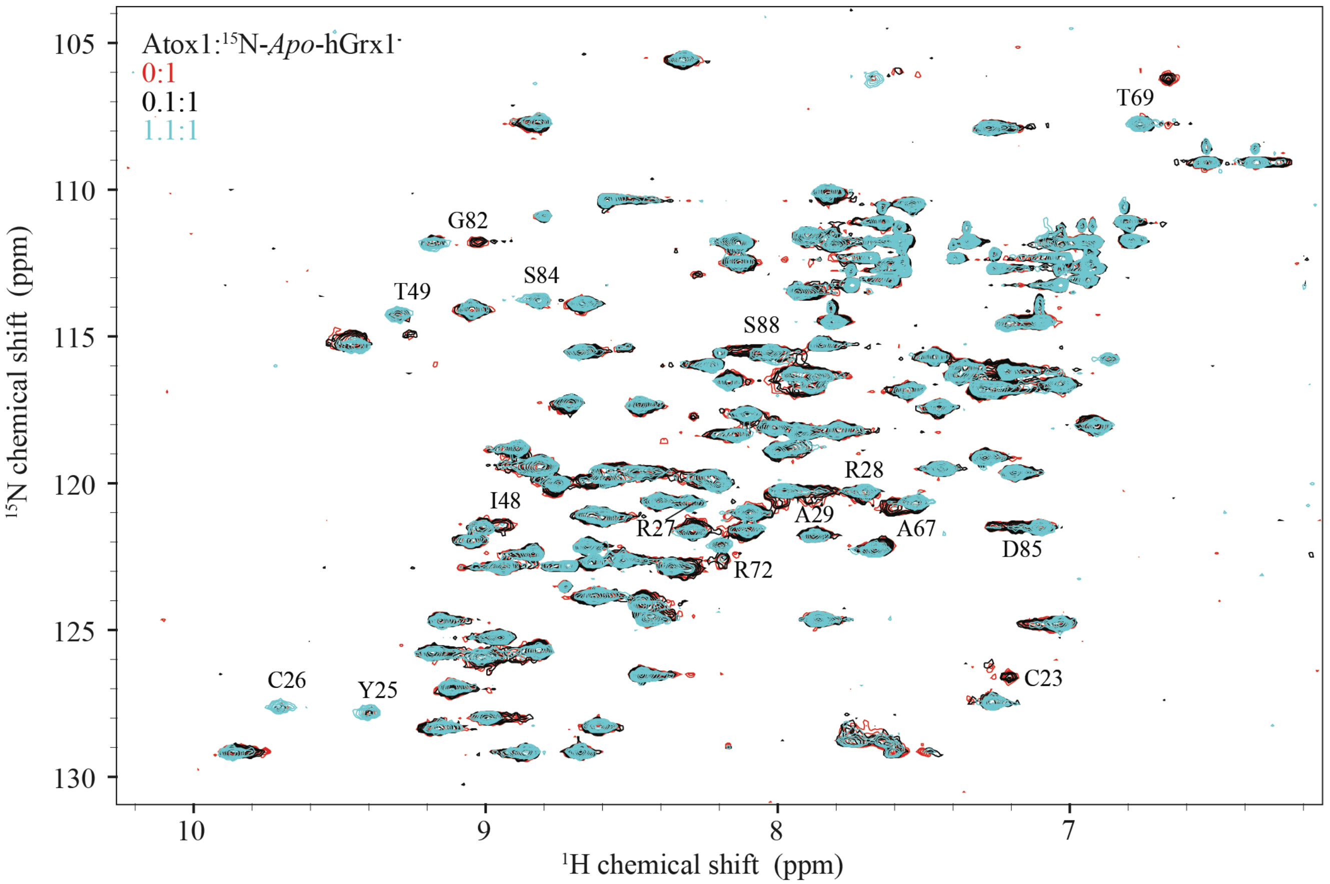
Atox1:15N-Apo-hGrx1
0:1
0.1:1
1.1:1
T69
G82
S84
T49
S88
R28
I48
A29
R27
A67
D85
R72
C26
Y25
C23
105
110
115
120
125
130
15N chemical shift (ppm)
10
9
8
7
1H chemical shift (ppm)

Figure S8A.

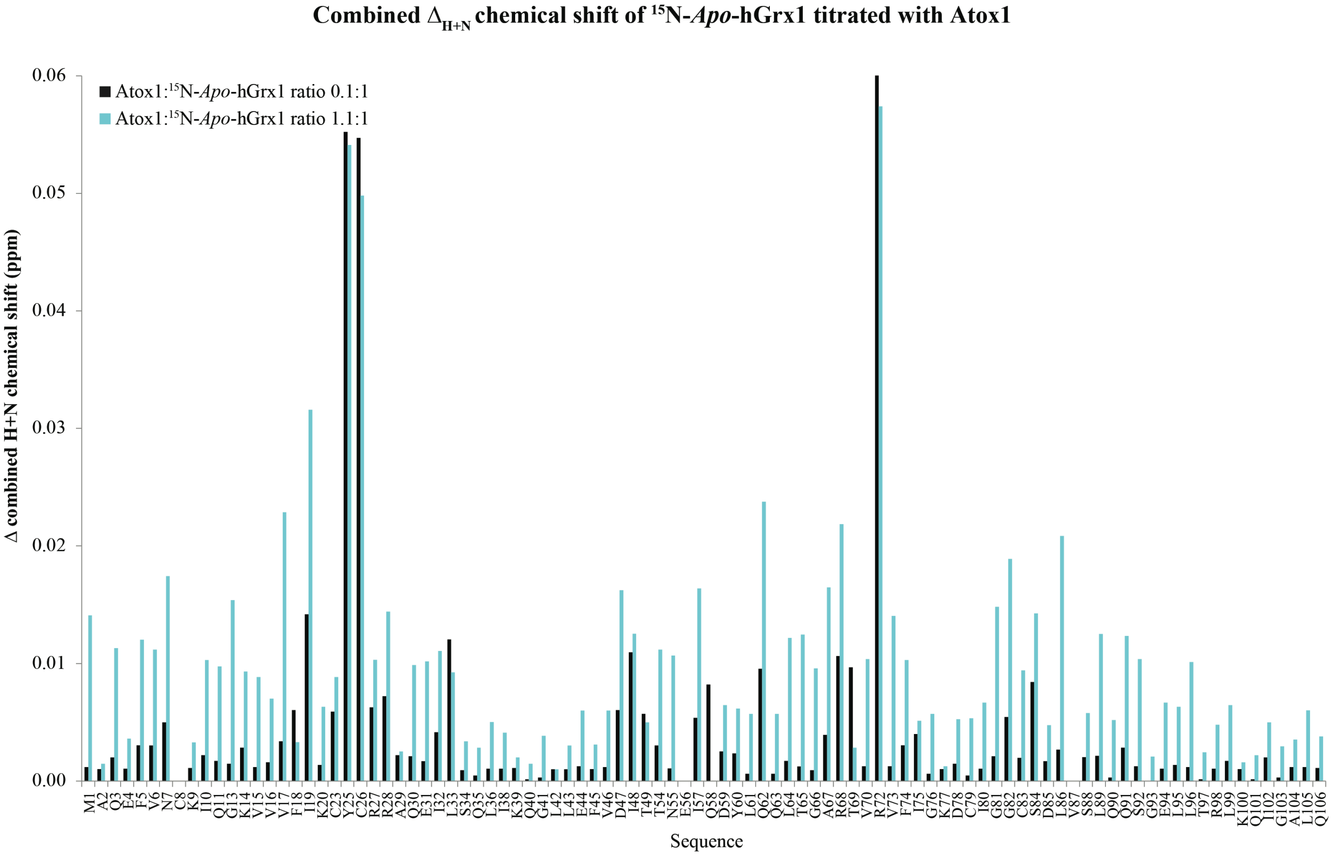

Figure S8B.

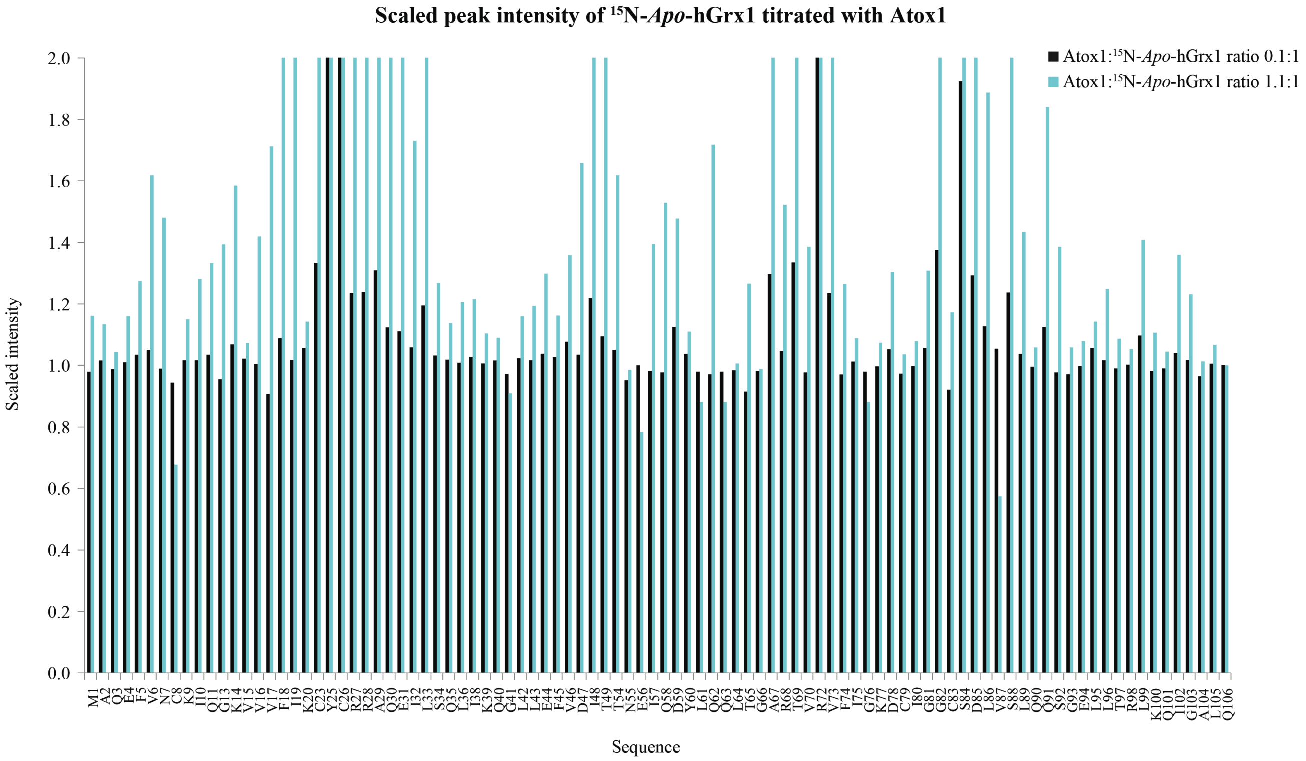

Figure S8C.

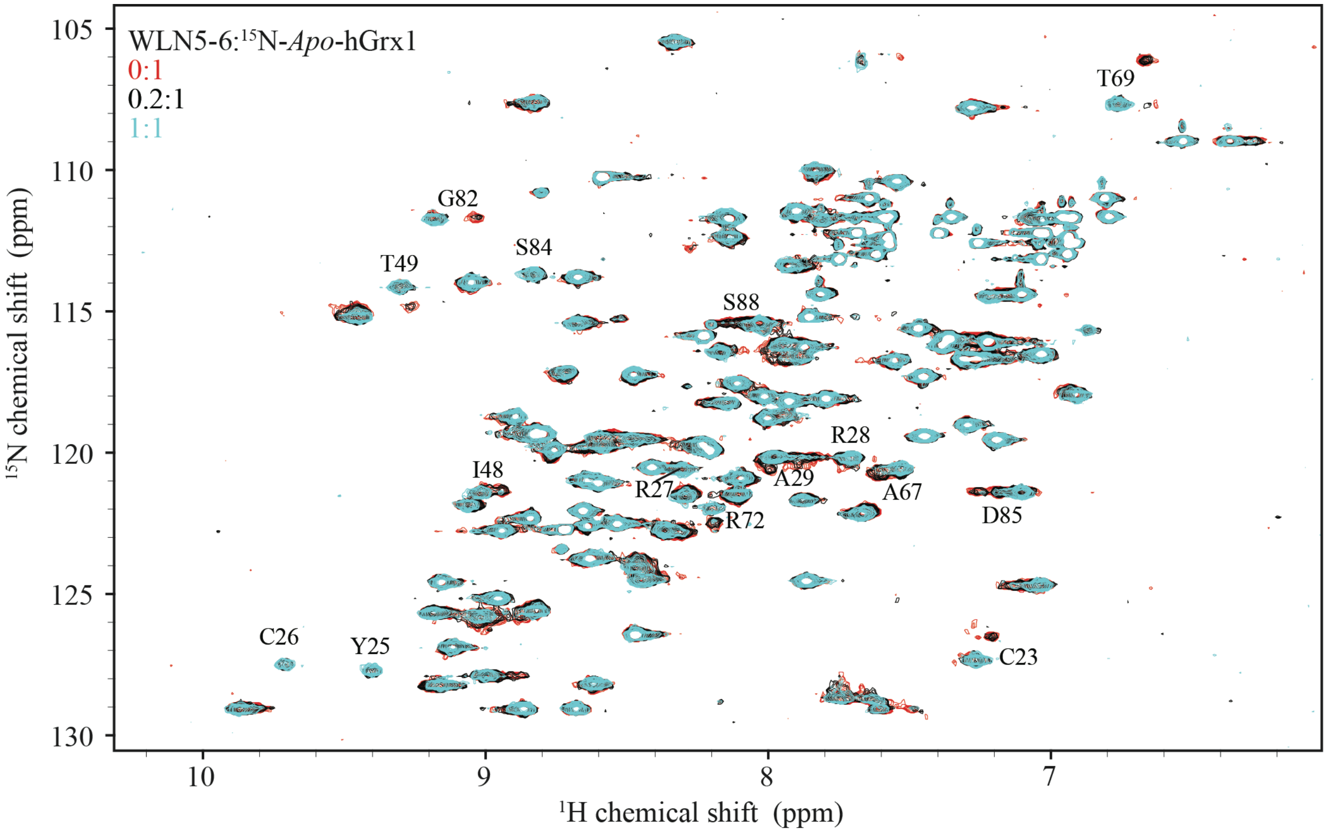
WLN5-6:15N-Apo-hGrx1
0:1
0.2:1
1:1
T69
G82
S84
T49
S88
R28
I48
R27
A29
A67
R72
D85
C26
Y25
C23
105
110
115
120
125
130
10
9
8
7
15N chemical shift (ppm)
1H chemical shift (ppm)

Figure S9A.

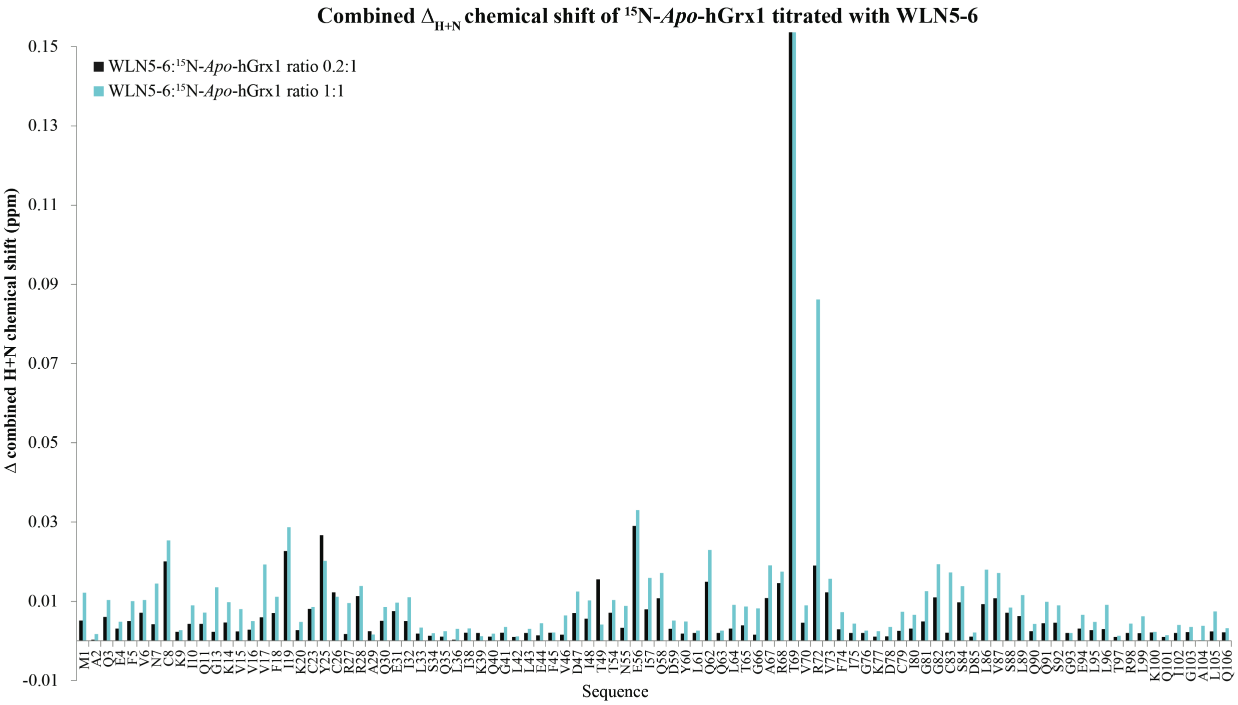

Figure S9B.

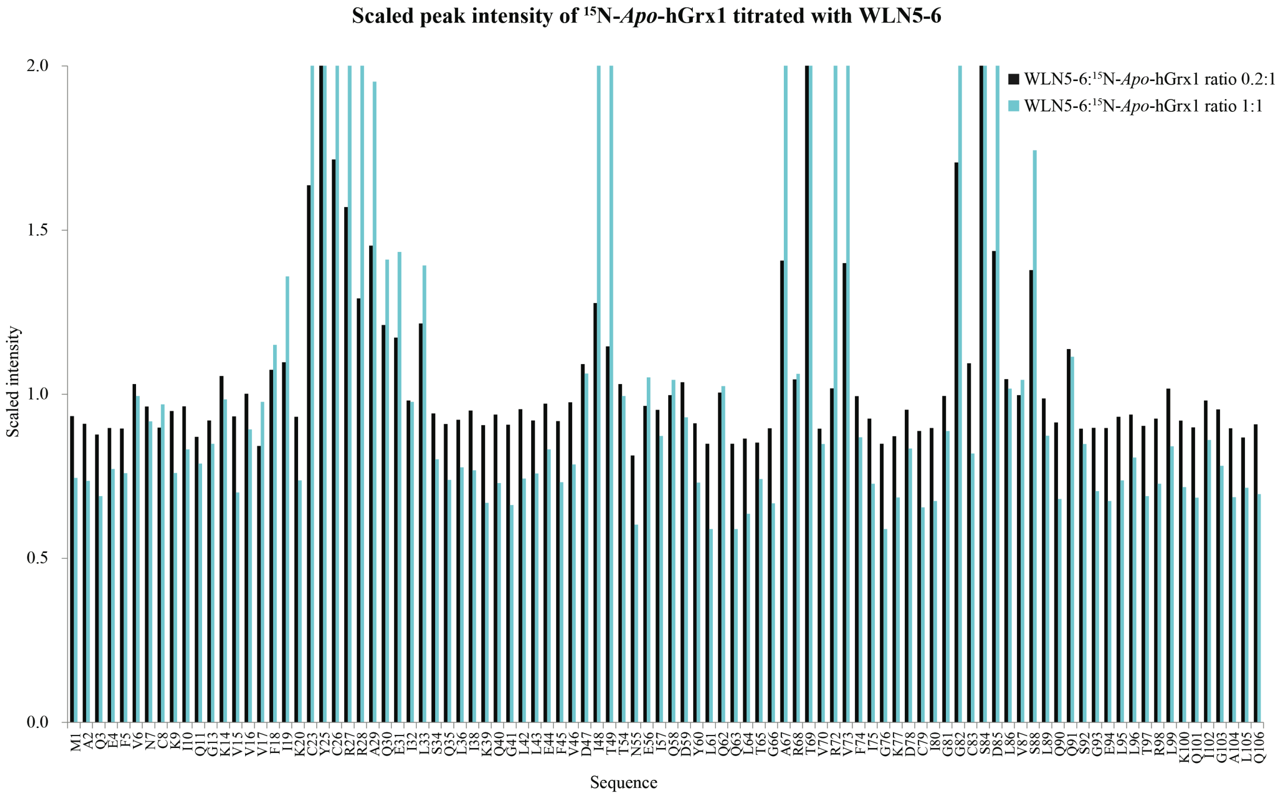

Figure S9C.

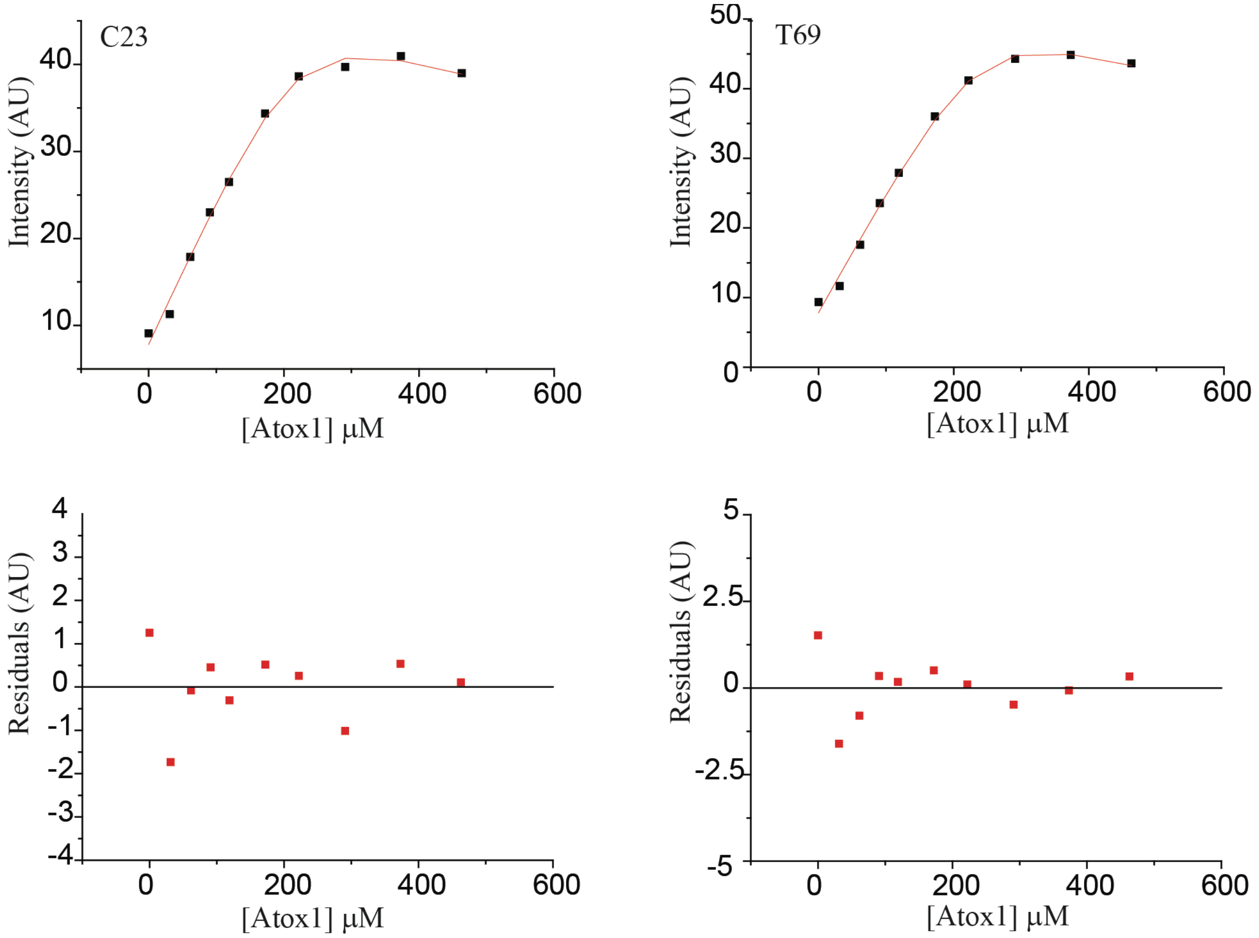

Figure S10A.

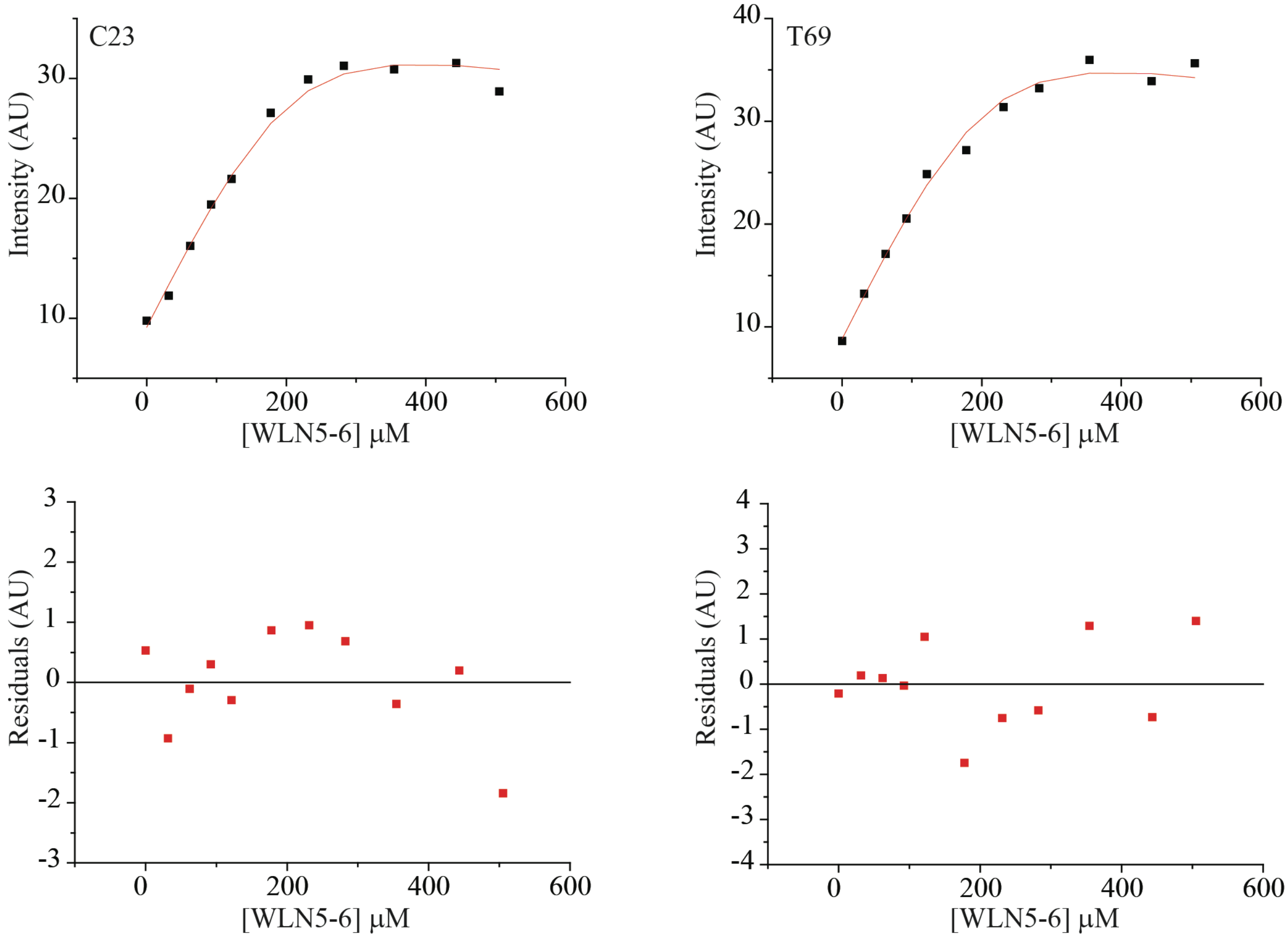
C23
Intensity (AU)
[WLN5-6] μM
T69
Intensity (AU)
[WLN5-6] μM
Residuals (AU)
[WLN5-6] μM
Residuals (AU)
[WLN5-6] μM

Figure S10B.

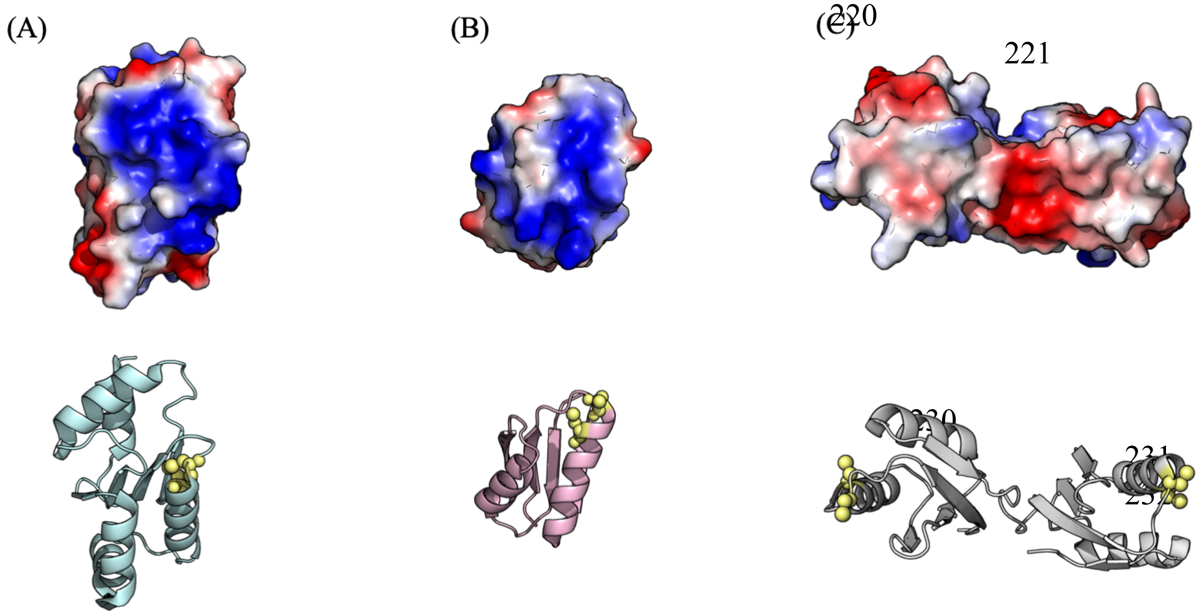

Figure S11.
